# MERTK activation drives osimertinib resistance in *EGFR*-mutant non–small cell lung cancer

**DOI:** 10.1172/JCI150517

**Published:** 2022-08-01

**Authors:** Dan Yan, Justus M. Huelse, Dmitri Kireev, Zikang Tan, Luxiao Chen, Subir Goyal, Xiaodong Wang, Stephen V. Frye, Madhusmita Behera, Frank Schneider, Suresh S. Ramalingam, Taofeek Owonikoko, H. Shelton Earp, Deborah DeRyckere, Douglas K. Graham

**Affiliations:** 1 Aflac Cancer and Blood Disorders Center, Children’s Healthcare of Atlanta and Department of Pediatrics, Emory University School of Medicine, Atlanta, Georgia, USA.; 2 Center for Integrative Chemical Biology and Drug Discovery, Division of Chemical Biology and Medicinal Chemistry, Eshelman School of Pharmacy, University of North Carolina at Chapel Hill, Chapel Hill, North Carolina, USA.; 3 Biostatistics and Bioinformatics Shared Resources, Winship Cancer Institute, Emory University School of Medicine, Atlanta, Georgia, USA.; 4 Department of Medicine, UNC Lineberger Comprehensive Cancer Center, Chapel Hill, North Carolina, USA.; 5 Department of Pathology,; 6 Winship Cancer Institute, and; 7 Department of Hematology and Medical Oncology, Emory University School of Medicine, Atlanta, Georgia, USA.; 8 Department of Pharmacology, School of Medicine, University of North Carolina at Chapel Hill, Chapel Hill, North Carolina, USA.

**Keywords:** Cell Biology, Oncology, Drug therapy, Lung cancer, Protein kinases

## Abstract

Acquired resistance is inevitable in non–small cell lung cancers (NSCLCs) treated with osimertinib (OSI), and the mechanisms are not well defined. The MERTK ligand GAS6 promoted downstream oncogenic signaling in *EGFR*-mutated (*EGFR^MT^*) NSCLC cells treated with OSI, suggesting a role for MERTK activation in OSI resistance. Indeed, treatment with MRX-2843, a first-in-class MERTK kinase inhibitor, resensitized GAS6-treated NSCLC cells to OSI. Both GAS6 and EGF stimulated downstream PI3K/AKT and MAPK/ERK signaling in parental cells, but only GAS6 activated these pathways in OSI-resistant (OSIR) derivative cell lines. Functionally, OSIR cells were more sensitive to MRX-2843 than parental cells, suggesting acquired dependence on MERTK signaling. Furthermore, MERTK and/or its ligands were dramatically upregulated in *EGFR^MT^* tumors after treatment with OSI in both xenograft models and patient samples, consistent with induction of autocrine/paracrine MERTK activation. Moreover, treatment with MRX-2843 in combination with OSI, but not OSI alone, provided durable suppression of tumor growth in vivo, even after treatment was stopped. These data identify MERTK as a driver of bypass signaling in treatment-naive and *EGFR^MT^*-OSIR NSCLC cells and predict that MRX-2843 and OSI combination therapy will provide clinical benefit in patients with *EGFR^MT^* NSCLC.

## Graphical abstract

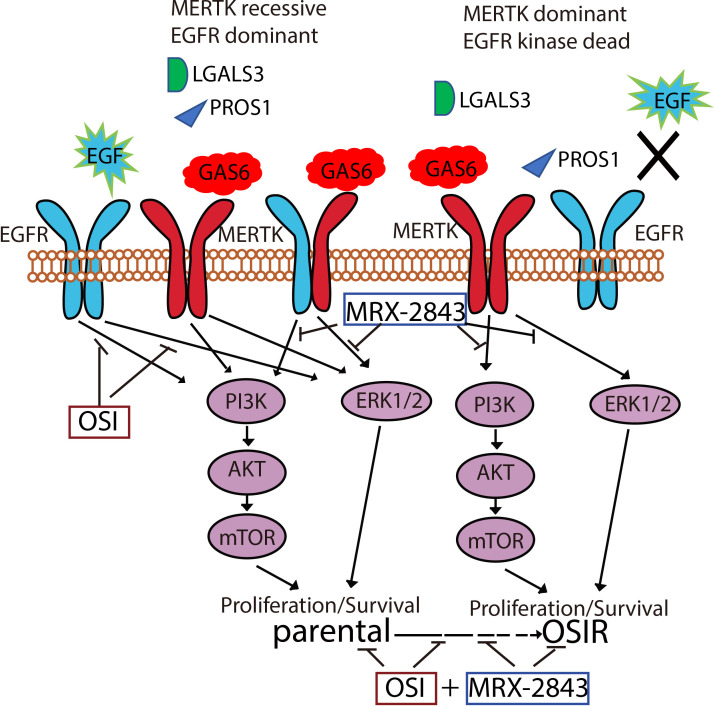

## Introduction

Lung cancer remains a global problem, causing more deaths in both men and women than any other cancer worldwide. Although the 5-year survival rate for lung cancer patients with localized disease is 59%, 5-year survival for all patients is only 21% due to the large proportion (57%) diagnosed at metastatic stage, with a 6% 5-year survival rate (1). Targeted biological agents and immune modulators have improved outcomes, but additional therapeutic approaches (possibly combining biologically targeted agents) are necessary for further enhancing patient survival.

EGFR is one of four members of the erbB family of RTKs. Somatic *EGFR* kinase domain mutations (*EGFR^MT^*) are detected in 10%–20% and 30%–60% of White and Asian non–small cell lung cancer (NSCLC) patients, respectively (2–5), leading to constitutive EGFR kinase activation (6). Stimulation with EGF or EGF-related peptide growth factors further enhances EGFR homo- and/or heterodimerization (7), resulting in autophosphorylation at multiple sites. EGFR-Y1068 autophosphorylation provides docking sites for SH2-containing adaptor proteins to directly activate the PI3K/AKT pathway (8), which maintains cell survival. Phosphorylation of EGFR on Y992, Y1148, and Y1173 sites results in recruitment of Shc to activate the MAPK/ERK pathway (9), which promotes cell proliferation.

During the last two decades, the therapeutic landscape for *EGFR^MT^* NSCLC patients has profoundly changed with the discovery that NSCLCs with *EGFR* mutations, either small, in-frame deletions or amino acid substitutions clustered around the ATP-binding pocket of the EGFR tyrosine kinase domain (10), show meaningful responses to EGFR tyrosine kinase inhibitors (TKIs), including gefitinib, erlotinib, and afatinib (11, 12). However, complete response to these early generation EGFR TKIs is not common and most tumors regrow during treatment with early generation EGFR TKIs, with a median time to progression of 9 to 13 months after initial response. In 60% of cases, disease progression is due to a secondary T790M “gatekeeper” mutation in the *EGFR* ATP-binding site (13–15). These findings led to the development of osimertinib (OSI)(16), a third-generation irreversible EGFR TKI targeting both activating mutations (such as the most common L858R and chromosome 19 deletions) and T790M mutations. The response rate for T790M-positive NSCLCs is more than 60% when treated with OSI (17). Based on these findings, OSI received fast-track FDA approval in 2015 for treatment of advanced NSCLC with T790M mutation and was later approved as a front-line agent for newly diagnosed NSCLC harboring activating mutations with or without T790M mutation due to superior efficacy relative to earlier generation EGFR TKIs gefitinib or erlotinib (progression-free survival [PFS] 18.9 versus 10.2 months) (18). More recently, the FLAURA clinical trial showed enhanced overall survival (OS) in patients with *EGFR^MT^* NSCLC treated with OSI (38.6 months) relative to comparator groups (31.8 months) (19), establishing OSI as the preferred treatment choice for these patients.

Similarly to the clinical experience with first-generation inhibitors, OSI resistance is now a growing clinical challenge, and it is critical to further understand resistance mechanisms. OSI covalently interacts with a conserved cysteine residue in EGFR (Cys797) (16), and *EGFR*-C797S mutation is the most frequent resistance-conferring mutation, detected in 7% of OSI-resistant (OSIR) patients (20). Other *EGFR* mutations, including L692V, E709K, L718Q, G724S, L792F, L792H, F795C, C797G, L798I, and L798V, have also been identified in OSIR tumors (21, 22). Alternatively, TKI resistance can occur through a bypass mechanism that reestablishes activation of key downstream signals to promote tumor cell survival and/or proliferation despite sustained inhibition of the original target. In this case, simultaneous inhibition of the original driver oncogene and secondary bypass signaling can be beneficial (22, 23). Despite these insights, the specific OSI resistance mechanisms remain to be elucidated in approximately two-thirds of patients (24). In the current study, we provide evidence that signaling through MERTK RTK can contribute to OSI resistance in a subset of NSCLCs.

MERTK is a member of the TAM (TYRO3, AXL, and MERTK) family RTKs that is overexpressed or ectopically expressed in about 70% of NSCLCs and is an attractive biologic target for treatment of NSCLC (25, 26). Activation by phosphatidyl serine (PtdSer) externalized on apoptotic cells complexed with the protein ligands protein S1 (PROS1) or growth arrest specific factor 6 (GAS6) leads to activation of downstream pathways, including PI3K/AKT and MAPK/ERK, and promotes tumor cell survival (27, 28). MERTK inhibition using shRNA or a MERTK kinase inhibitor reduced NSCLC colony formation in vitro and xenograft tumor growth in vivo (25, 29). Exogenous overexpression of MERTK in erlotinib-sensitive cells induced resistance to erlotinib, and addition of a MERTK inhibitor resensitized these erlotinib-resistant cells to erlotinib treatment (30), demonstrating a role for MERTK as a mediator of resistance to EGFR TKIs. However, it is not known whether MERTK overexpression and/or activation can drive OSI resistance. Here, we show that development of OSI resistance in preclinical models correlates with induction of MERTK and ligand expression and that combined treatment with OSI and a MERTK inhibitor already in human phase I trials is effective in reversing OSI resistance (ClinicalTrials.gov, NCT04762199).

## Results

### OSI does not eradicate PI3K/AKT and MAPK/ERK signaling in OSIR cells.

**Figure 1:**
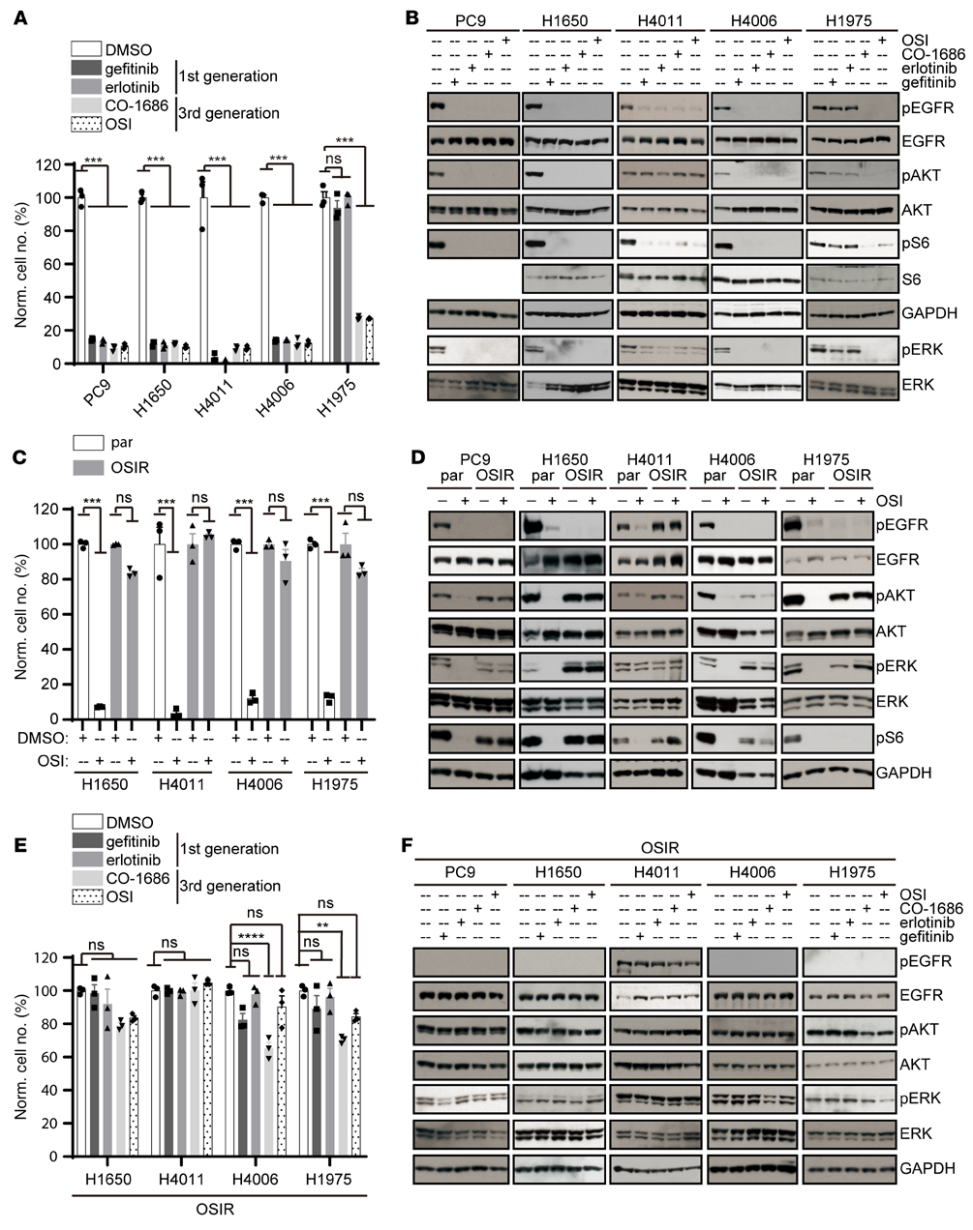
Activation of PI3K/AKT and MAPK/ERK pathways is independent of EGFR activation in OSIR cell-line derivatives with cross resistance to other EGFR TKIs. (**A**, **C**, and **E**) Nuclight Red–labeled *EGFR^MT^* NSCLC cell lines and OSIR derivatives were treated with 1 μM of gefitinib, erlotinib, CO-1686, or OSI for 3 to 4 days, and cell numbers relative to vehicle-treated control (DMSO) were determined using the Incucyte ZOOM Live Cell Imaging System. Mean values and standard errors derived from 3 independent experiments are shown.**P < 0.01; ***P < 0.001, ****P < 0.0001; 1-way ANOVA; Norm= cell numbers were normalized to cells treated with DMSO. (**B**, **D**, and **F**) Cells were serum starved overnight and then treated with 1 μM of EGFR TKIs or DMSO for 2 hours and phosphorylated (denoted by p); total proteins were assessed by immunoblot. Images shown are representative of 3 independent experiments. See complete unedited blots in the supplemental material.

NSCLC cell lines carrying either in-frame *EGFR* deletions (E746-A750del in PC9 and H4006; K745-E749del+A750K in H1650) or a site-specific *EGFR* mutation (L858R mutation in H4011) were sensitive to treatment with the first-generation EGFR TKIs gefitinib and erlotinib, as expected, as indicated by decreased expansion over 72 hours in culture (Figure 1A). EGFR activation and downstream signaling through the PI3K/AKT-S6 and MAPK/ERK pathways were consistently inhibited in the presence of OSI (Figure 1B), despite a lack of AKT phosphorylation inhibition in H4011 cells in response to OSI, which has been reported previously (31). *EGFR* T790M mutation accounts for about 60% of acquired resistance to first-generation EGFR TKIs (14, 15), and indeed, cell expansion and downstream signaling were not significantly affected in cultures of the H1975 cell line, which carries both *EGFR* L858R and T790M mutations, treated with 1 μM gefitinib or erlotinib, demonstrating resistance (Figure 1, A and B). In contrast, treatment with the third-generation EGFR TKIs OSI or CO-1686 inhibited cell expansion in all *EGFR^MT^* cell lines, irrespective of T790M mutation status (Figure 1A). Phosphorylation at EGFR-Y1068, an important docking site for SH2-containing adaptor proteins to directly activate PI3K/AKT (8), was also diminished in the presence of OSI or CO-1686 (Figure 1B). OSIR cell lines were established by gradually escalating the concentration of OSI from 10 nM to 3 μM. Cell expansion was not reduced in cultures of OSIR cell line derivatives treated with 1 μM OSI, confirming OSI resistance (Figure 1C). Phosphorylated EGFR (pEGFR) was not detected in 4 out of 5 OSIR cell lines tested (even in the absence of OSI) (Figure 1D). However, PI3K/AKT and MAPK/ERK signaling were continuously activated in the presence of OSI or CO-1686, indicating activation of critical oncogenic pathways by an alternative mechanism in these OSIR cells.

To determine whether acquired *EGFR* mutations contribute to OSI resistance in these cell lines, the *EGFR* open-reading frame was sequenced. However, no additional *EGFR* mutations were detected. Further, it has been reported that C797S mutations that confer resistance to OSI resensitize EGFR to earlier generation EGFR TKIs (32). However, treatment with erlotinib or gefitinib did not inhibit expansion of the OSIR cell lines described here (Figure 1E) or block downstream signaling (Figure 1F). Thus, these OSIR cells were cross-resistant to other EGFR TKIs.

### MERTK activation drives bypass signaling in OSIR EGFR^MT^ NSCLC cells.

**Figure 2:**
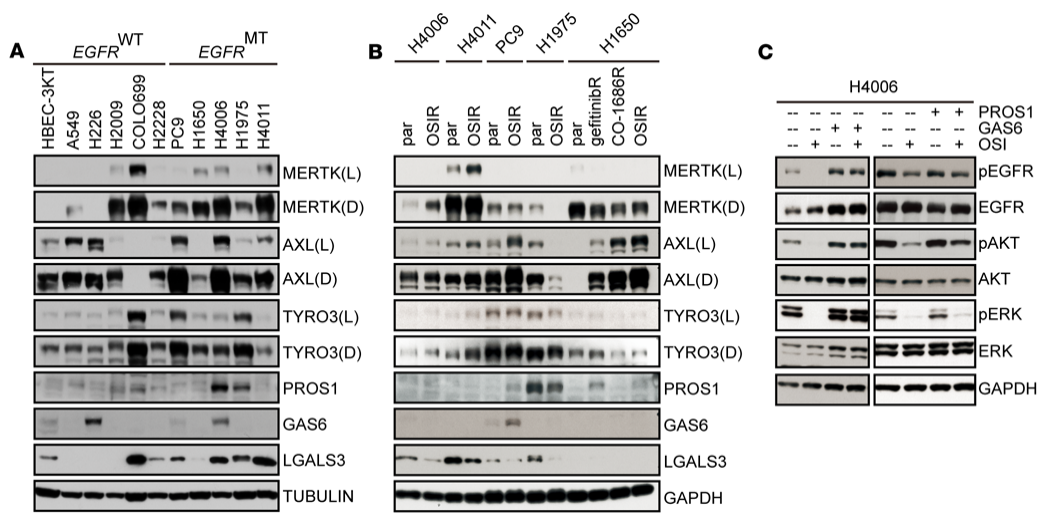
MERTK drives oncogenic signaling in the presence of OSI. (**A** and **B**) Cell lysates were prepared from the indicated cell lines and TAM receptors, and their ligands were assessed by immunoblot. (**A**) *EGFR^WT^*, *EGFR^MT^*, and HBEC-3KT immortalized normal human bronchial epithelial cell lines. (**B**) OSIR, parental, gefitinib-resistant (gefitinibR), and CO-1686–resistant (CO-1686R) cell lines. (**C**) H4006 cells were serum starved overnight and then treated with DMSO or 100 nM OSI in serum-free medium for 2 hours followed by 10 minutes of stimulation with 50 nM GAS6, 50 nM PROS1, or vehicle and phosphorylated; total proteins were detected by immunoblot. Images shown are representative of 3 independent experiments. See complete unedited blots in the supplemental material.

TAM receptors and their ligands (PROS1, GAS6, and LGALS3) were frequently expressed on NSCLC cell lines (Figure 2A). In contrast to AXL and TYRO3, MERTK was not expressed on HBEC3-KT, an immortalized normal human bronchial cell line. Interestingly, MERTK was upregulated in 2 out of 5 OSIR cell lines (H4006 and H4011) relative to parental controls (Figure 2B). To determine whether MERTK overexpression was sufficient to confer OSI resistance, H4006 cells with stable MERTK overexpression (H4006-MERTK) were established (Supplemental Figure 1A; supplemental material available online with this article; https://doi.org/10.1172/JCI150517DS1). Overexpressed MERTK was functional, as indicated by increased levels of phosphorylated MERTK following stimulation with GAS6 relative to H4006 control cells with empty vector (H4006-vector) (Supplemental Figure 1B). However, H4006-MERTK cells retained sensitivity to OSI treatment and were indistinguishable from H4006-vector cells in colony-forming assays (Supplemental Figure 1C). Thus, MERTK overexpression alone was not sufficient to provide OSI resistance. However, in the presence of the MERTK ligands GAS6 or PROS1, downstream signaling through pAKT and/or pERK was no longer inhibited by OSI (Figure 2C and Supplemental Figure 1D), suggesting that activated MERTK mediates bypass signaling through these pathways and may thereby contribute to OSI resistance in this context. TYRO3 was also upregulated in H4006-OSIR and H4011-OSIR cells relative to parental cell lines, and AXL was upregulated in the PC9 and H1650 OSIR cell lines, consistent with other reports of AXL upregulation in OSIR cells (Figure 2B) (31). Similarly, PROS1 and GAS6 ligands were upregulated in a subset of OSIR cell lines.

### MERTK has differential roles and interactions with EGFR in parental and OSIR cells.

**Figure 3:**
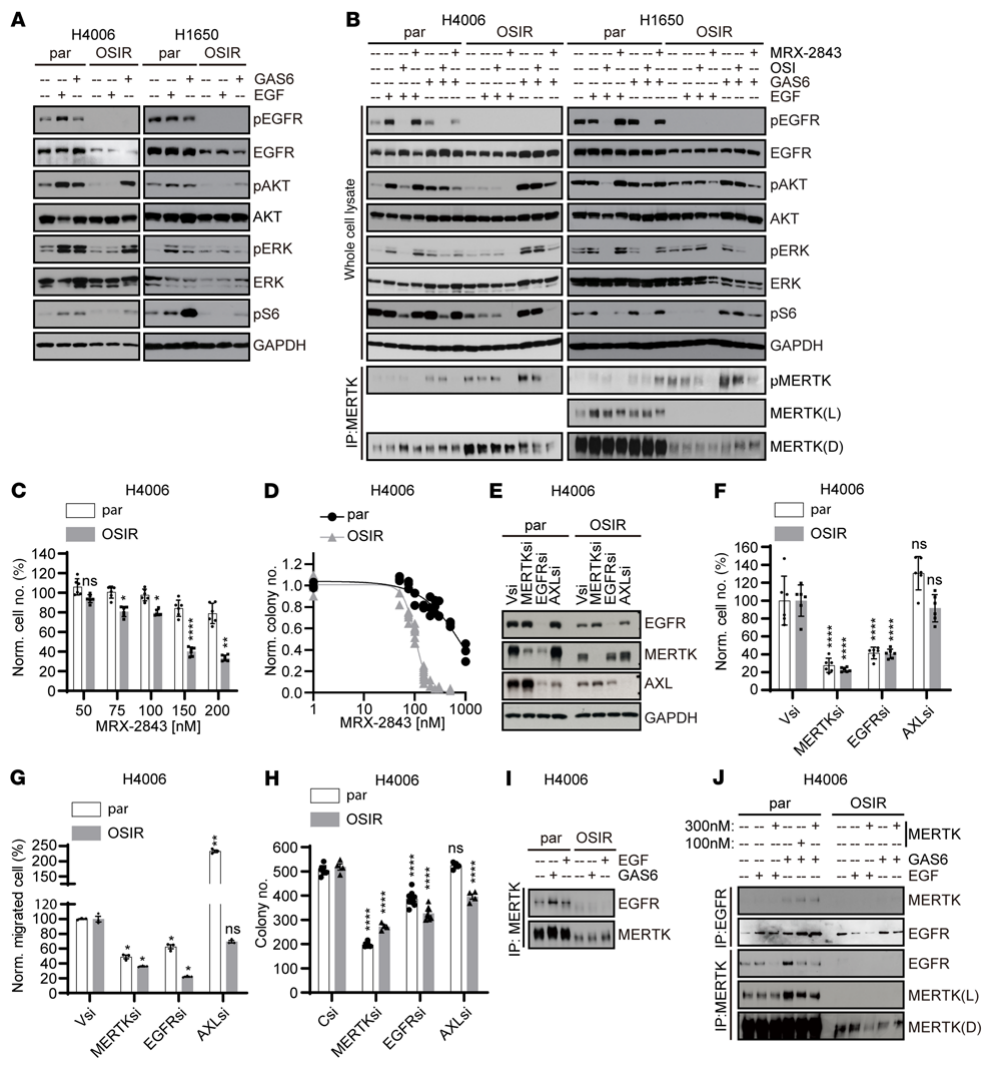
MERTK drives oncogenic signaling in OSIR cells and associates with EGFR in parental but not OSIR cells. (**A** and **B**) OSIR or parental cells were serum starved overnight and then stimulated with GAS6 or EGF ligands for 10 minutes. Phosphorylated and total proteins were assessed by immunoblot. (**B**) Cultures were treated with 1 μM OSI or 300 nM MRX-2843 for 2 hours before ligand stimulation. MERTK was detected after immunoprecipitation. (**C**) Nuclight Red–expressing H4006-par or H4006-OSIR cells were treated with MRX-2843 for approximately 100 hours, and cell numbers were counted using the Incucyte ZOOm Live Cell Imaging System. Cell numbers relative to vehicle are shown (*n* = 6). (**D**) H4006-par and H4006-OSIR cells were cultured at low density and treated with MRX-2843 for 7 to 8 days before colonies were stained and counted. Colony numbers relative to vehicle are shown. The sigmoid shown was derived using a 4-parameter variable-slope nonlinear regression model. (**E**–**H**) Nuclight Red–expressing H4006-par or H4006-OSIR cells were transfected with siRNA against *MERTK*, *EGFR*, or *AXL* or with nontargeting siRNA (Vsi) for 24 hours and then cultured for assessment of (**F**) cell expansion over 80 to 90 hours, (**G**) chemotaxis, and (**H**) colony formation. (**E**) Protein levels were determined by immunoblot 24 hours after transfection to confirm target knockdown. (**I** and **J**) Serum-starved H4006-par and H4006-OSIR cells were stimulated with GAS6 or EGF ligand for 10 minutes and treated with pervanadate; then MERTK or EGFR was immunoprecipitated from cell lysates and EGFR and MERTK proteins were detected by immunoblot. (**J**) Cultures were treated with MRX-2843 or vehicle for 2 hours before ligand stimulation. Representative immunoblots from 3 independent experiments are shown. Mean values and standard errors were derived from 3 to 8 independent experiments. *P < 0.05; **P < 0.01; ****P < 0.0001, 1-way ANOVA. See complete unedited blots in the supplemental material.

Although MERTK was upregulated in the H4006 and H4011 cell lines during acquisition of OSI resistance, overexpression of MERTK was not sufficient to provide bypass signaling in the absence of ligand, suggesting that MERTK activation, rather than expression level, drives OSI resistance. Thus, MERTK may mediate bypass signaling in OSIR cell lines even when MERTK is not upregulated. To evaluate the molecular mechanisms by which MERTK may mediate OSI resistance in both contexts, one cell line that spontaneously upregulated MERTK during acquisition of OSI resistance (H4006) and one that did not (H1650) were chosen for further study. Downstream signaling pathways were analyzed in *EGFR^MT^* parental and OSIR derivative cell lines, and differential responses to stimulation with GAS6 and EGF ligands were noted. Treatment with either EGF or GAS6 enhanced downstream PI3K/AKT and MAPK/ERK signaling in parental *EGFR^MT^* H4006 and H1650 cells (Figure 3A), indicating that both EGFR and TAM RTKs are upstream of these pathways. In contrast, pEGFR was not detected and PI3K/AKT and MAPK/ERK pathways were not enhanced following treatment with EGF in OSIR cells (Figure 3A), indicating that EGFR signaling was dysfunctional in OSIR cells. However, MERTK phosphorylation was increased in H4006-OSIR and H1650-OSIR cells compared with parental cells (Figure 3B), PI3K/AKT and MAPK/ERK pathways were activated following GAS6 stimulation (Figure 3A), and activation of these pathways was reduced upon addition of a MERTK kinase inhibitor, MRX-2843 (Figure 3B) (33, 34), implicating MERTK as a critical mediator of oncogenic signaling in OSIR cells. In contrast, treatment with MRX-2843 was not sufficient to diminish PI3K/AKT and MAPK/ERK signaling in parental cells (Figure 3B), suggesting a preferential role for MERTK signaling in OSIR cells. Indeed, H4006-OSIR cells were more sensitive to MRX-2843 than parental cells in cell expansion (EC_50_=219.4 nM [198.3–242.9] for H4006 and EC_50_=126.6 nM [106.9–149.9] for H4006-OSIR, respectively) (Figure 3C) and colony formation assays (EC_50_=676.3 nM [506.6-1108] for H4006 and EC_50_=102.9 nM [97.15–109.1] for H4006-OSIR, respectively) (Figure 3D).

Additional differences in the interplay between MERTK and EGFR were observed in OSIR and parental cell lines. EGFR knockdown led to decreased MERTK expression in parental cells, but not in OSIR cells (Figure 3E). Moreover, MERTK and EGFR coimmunoprecipitated from parental cell lysates, but not from OSIR cell lysates (Figure 3I), and the interaction in parental cells was further enhanced following stimulation with either GAS6 or EGF (Figure 3, I and J). Conversely, addition of MRX-2843 decreased association of EGFR with MERTK (Figure 3J). Thus, the interaction between MERTK and EGFR in parental cells was MERTK kinase dependent.

We next evaluated roles for MERTK, EGFR, and AXL in NSCLC tumor cell expansion, migration, and colony formation using siRNA-mediated knockdown (Figure 3E). Knockdown of either EGFR or MERTK significantly inhibited cell expansion (Figure 3F), migration (Figure 3G), and colony formation (Figure 3H) in both H4006-parental (H4006-par) and H4006-OSIR cells. AXL has been previously implicated as a mediator of OSI resistance (31), and AXL knockdown in H4006-OSIR cells inhibited colony formation (Figure 3H), but did not affect cell expansion (Figure 3F). In contrast to a previous report demonstrating decreased chemotactic migration in colorectal cancer cells with AXL knockdown (35), AXL knockdown promoted chemotactic migration in H4006-par cells and had no significant effect on migration in H4006-OSIR cells (Figure 3G).

### L593 is a predicted MERTK-selective gatekeeper site for MRX-2843.

MRX-2843 is a potent ATP-competitive dual MERTK and FLT3 TKI that is predicted to have off-target activity against a limited number of other kinases (TRKA, AXL, and LOK) in cell-based assays (33, 34). To determine whether MRX-2843–mediated inhibition of MERTK and EGFR interaction in parental cells was an “on-target” effect, we created an inhibitor-resistant but catalytically active MERTK mutant designed through a comparative structural analysis of 3D structures of compound protein complexes. A docking model of MRX-2843 to the crystal structure of MERTK in complex with adenosine-5′-diphosphate (ADP) (PDB ID: 3BRB) was used to determine potential sites to block drug docking and retain kinase activity. The number of close contacts between the compound and the protein was used as a metric for the likelihood that a residue might affect the compound binding. Accordingly, residues having a large number of close contacts with MRX-2843 and significantly fewer contacts with ADP would be the most promising mutation sites. Also, enzymes are highly dynamic systems allowing a compound to adopt a broad range of binding modes. To take into account this protein and ligand flexibility, the conformational space of MERTK in complex with MRX-2843 and ADP, respectively, was sampled through all-atom molecular dynamics (MD) simulations. To assess the number of close contacts for each compound-protein complex, we counted all ligand-residue atom pairs with interatomic distances less than 4.5 angstroms (Å) (50,000 complexes were extracted from each MD trajectory).

**Figure 4:**
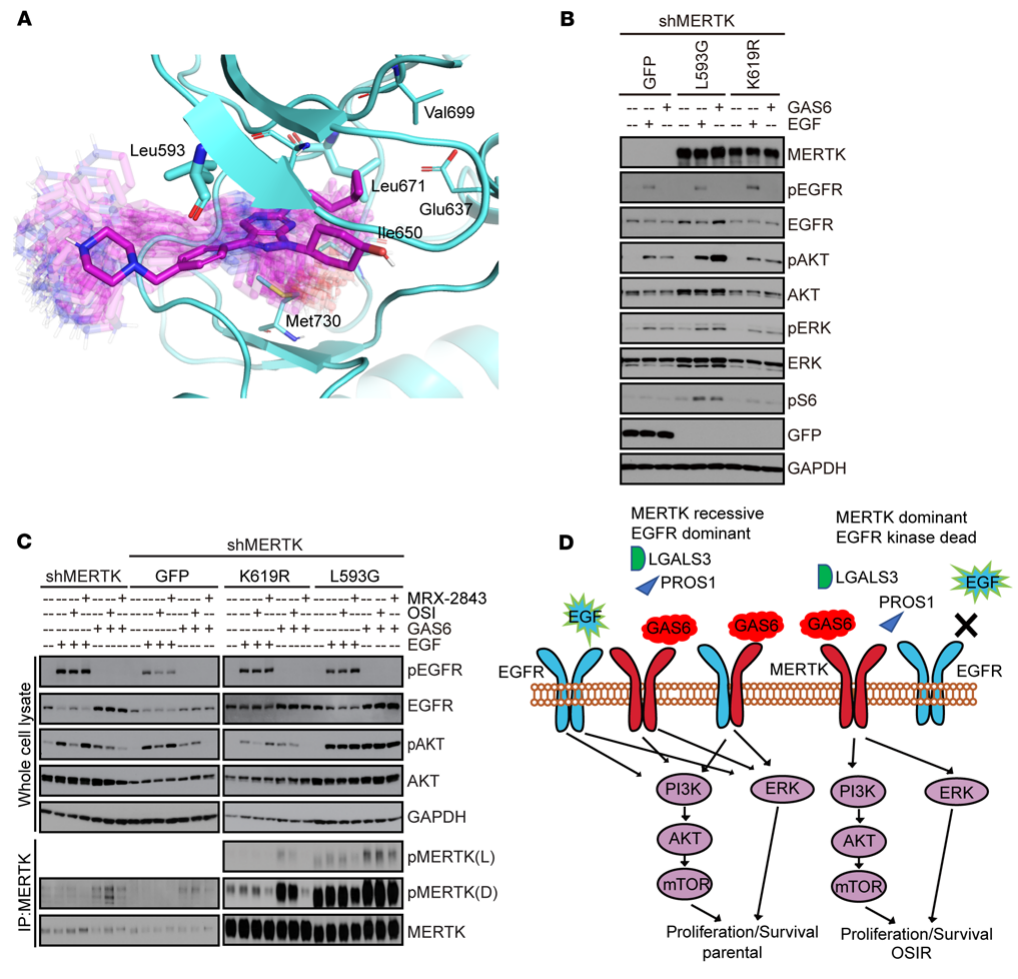
MERTK mediates downstream oncogenic signaling in NSCLC cells. (**A**) MERTK (cyan) in complex with MRX-2843 (magenta sticks). Representative conformations of the inhibitor sampled from MD simulations are shown in semitransparent rendering. Eight residues considered as potential mutation sites to block MRX-2843 binding yet preserve kinase activity and 6 visible residues are highlighted and rendered as cyan sticks. (**B** and **C**) 633 cells with shRNA-mediated inhibition of endogenous MERTK and ectopic expression of MERTK-L593G or MERTK-K619R mutant proteins or GFP control were serum starved overnight and then stimulated with GAS6 or EGF for 10 minutes. Phosphorylated and total proteins were detected by immunoblot. Images shown are representative of 3 independent experiments. (**C**) Cells were treated with 300 nM MRX-2843 or 1 μM OSI for 2 hours before stimulation with GAS6 or EGF. MERTK was detected following immunoprecipitation. (**D**) Model summarizing the proposed mechanisms by which MERTK shifts from its recessive role in OSI-sensitive *EGFR^MT^* NSCLC cells to a dominant role in OSIR *EGFR^MT^* NSCLC cells through differential interplay with EGFR.See complete unedited blots in the supplemental material.

A total of 19 residues (Lys591, Leu593, Gly594, Gly596, Glu597, Val601, Ala617, Lys619, Glu637, Ile650, Val669, Leu671, Pro672, Phe673, Met674, Gly677, Thr681, Met730, and Asp741) were detected in close proximity to MRX-2843. After removing those that use their backbone atoms to interact with the compound and residues that are critical for the structural integrity of the enzyme (Lys619 and Asp741), 8 residues (Leu593, Val601, Ala617, Glu637, Ile650, Val669, Leu671, Met730) were considered as potential mutation sites. These remaining residues were analyzed for their close contacts with MRX-2843 and ADP in ligand-protein complexes sampled from the MD trajectories. We found that 5 of 8 residues had a larger number of close contacts with MRX-2843 than with ADP, although 2 of them, Glu637 and Val669, had very few contacts overall. Hence, only 3 residues remained as promising mutation sites: Leu671, Leu593, and Ile650. From these, we excluded Ile650, since it is already a natural “mutation” site within the TAM kinase family (Ala581 in TYRO3, Met599 in AXL), with no critical effect on drug or ligand binding. Therefore, Leu593 and Leu671 were computationally selected as potential mutation sites (Figure 4A), with L593G being the most likely to achieve selective inhibition of MRX-2843 binding, since this mutation would switch off all favorable Van der Waals interactions with the inhibitor. Thus, investigating this putative gatekeeper mutation could provide compelling evidence that MRX-2843 therapeutic activity is mediated via inhibition of MERTK kinase activity and not through an off-target kinase.

### MERTK L593G mutation blocks MERTK inhibition and downstream signaling in response to MRX-2843.

To evaluate whether the putative gatekeeper mutation blocks the effects of MRX-2843 in NSCLC cells, a derivative of the 633 NSCLC cell line stably expressing an shRNA targeting the untranslated region of MERTK (633-shMERTK) (36) was transduced with plasmids driving ectopic expression of the L593G predicted gatekeeper mutant protein, a kinase-dead K619R mutant protein (37), or GFP as a control (Figure 4B). Stimulation with GAS6 increased phosphorylation of AKT and S6 in 633-shMERTK cells expressing the L593G mutant compared with 633-shMERTK cells expressing GFP (Figure 4B), demonstrating that the L593G mutant protein retained MERTK kinase activity. In contrast, 633-shMERTK cells expressing the K619R mutant protein had downstream signaling similar to that of cells expressing GFP (Figure 4B), confirming that kinase activity was abrogated in the K619R mutant protein. Further, treatment with MRX-2843 inhibited MERTK phosphorylation in 633-shMERTK cells expressing the K619R mutant protein, presumably due to leftover endogenous MERTK, while MERTK phosphorylation in 633-shMERTK cells expressing the L593G mutant protein was not significantly inhibited (Figure 4C). Further, GAS6-dependent AKT phosphorylation was abolished with the addition of MRX-2843 in 633-shMERTK cells with GFP or K619R addback, but not with L593G addback. These data support the idea that *MERTK*-L593G is an inhibitor-resistant but catalytically active MERTK mutant and demonstrate signaling through AKT-S6 downstream of MERTK in 633 NSCLC cells.

Together, these data support a model (Figure 4D) in which EGFR and MERTK are both functional and interact with each other in parental cells (Figure 3, A and J). EGFR is a dominant driver of oncogenic signaling in this context, but in the presence of MERTK ligand, treatment with OSI is not sufficient to abrogate signaling (Figure 3B), providing a rationale for OSI and MRX-2843 combination therapy. In OSIR cells, EGFR no longer mediates downstream signaling and the cells are more dependent on MERTK kinase activity. MERTK and EGFR interaction was dramatically reduced in OSIR cells (Figure 3, I and J), and downstream signaling (Figure 3B), cell expansion (Figure 3C), and colony formation (Figure 3D) were more affected by MRX-2843 compared with parental cells, highlighting the potential benefit of inhibiting MERTK kinase activity in OSIR *EGF*R*^MT^* NSCLC.

### Treatment with MRX-2843 sensitizes OSIR cells to EGFR TKIs.

**Figure 5:**
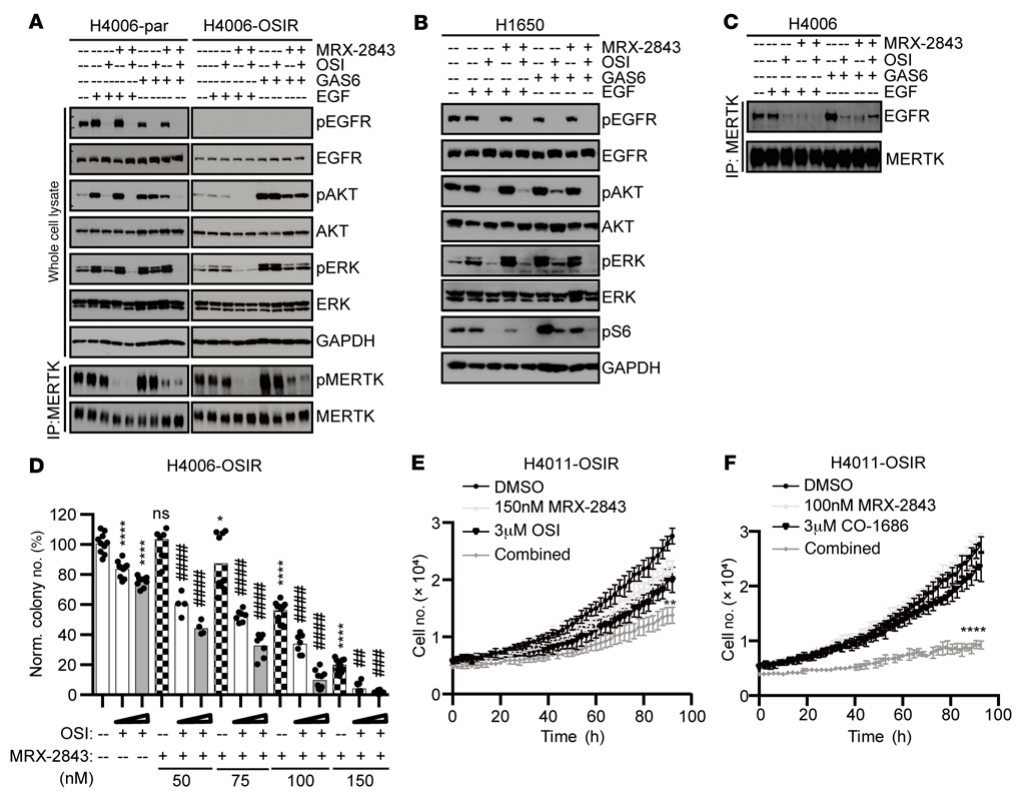
MRX-2843 sensitizes OSIR cell lines to EGFR TKIs. (**A**–**C**) Cells were serum starved overnight and then treated with 1 μM OSI and/or 100 nM MRX-2843 for 2 hours before stimulation with GAS6 or EGF for 10 minutes. Phosphorylated and total proteins were assessed by immunoblot. (**A** and **C**) Cultures were treated with pervanadate prior to preparation of cell lysates, and MERTK was immunoprecipitated before immunoblot. (**D**) H4006-OSIR cells were cultured at low density in the presence of the indicated concentrations of MRX-2843 and/or OSI (1 μM or 2 μM) or vehicle for 8 days before colonies were stained and counted. *P < 0.05 versus vehicle; ****P < 0.0001 versus vehicle; ^##^*P* < 0.01 versus single agents; ^###^*P* < 0.001 versus single agents; ^####^*P* < 0.0001 versus single agents, 1-way ANOVA. (**E** and **F**) Nuclight Red–expressing H4011-OSIR cells were treated with MRX-2843, CO-1686, or OSI alone or combined, and cell numbers were determined at 2-hour intervals using the Incucyte ZOOM Live Cell Imaging System. Images shown are representative of 3 independent experiments. **P < 0.01; ****P < 0.0001, 1-way ANOVA. Mean values and standard errors were derived from 4 to 10 (**D**) or 3 (**E** and **F**) independent experiments. See complete unedited blots in the supplemental material.

MERTK and EGFR share many downstream signaling pathways (28), and MERTK can mediate bypass signaling in the context of OSI resistance. Thus, we hypothesized that treatment with MRX-2843 would resensitize OSIR cells to OSI treatment. Indeed, AKT phosphorylation remained unchanged when H4006-par or H1650-par cells were treated with OSI in the presence of GAS6, but combined treatment with MRX-2843 and OSI abrogated AKT phosphorylation (Figure 5, A and B). Similarly, ERK phosphorylation was only partially reduced in response to OSI treatment in the presence of GAS6, but was undetectable in cells treated with OSI and MRX-2843 (Figure 5, A and B). Similarly to what is shown in Figure 3J, coprecipitation of EGFR with MERTK was enhanced in parental cells following GAS6 stimulation and was inhibited in the presence of either MRX-2843 or OSI (Figure 5C). Treatment with OSI also reduced this interaction, and combined treatment with OSI and MRX-2843 was more effective in this regard than treatment with either single agent in the presence of EGF (Figure 5C). In H4006-OSIR cells, treatment with MRX-2843 reduced downstream oncogenic signaling (Figure 5A) and inhibited colony formation in a dose-dependent manner (Figure 5D). Moreover, treatment with MRX-2843 and OSI combined reduced colony formation even further (Figure 5D). Similarly, treatment with MRX-2843 and OSI or CO-1686 reduced cell expansion more effectively than monotherapies in H4011-OSIR cell cultures (Figure 5, E and F). Thus, combined MRX-2843 and OSI provided enhanced therapeutic benefit relative to monotherapies in OSIR cell cultures.

Previous studies identified AXL as a mediator of resistance to EGFR TKIs, including OSI (21, 22, 31). To investigate whether AXL can play a similar role in cell lines with MERTK-mediated bypass signaling, studies were conducted using the clinically relevant AXL inhibitor R428 (bemcentinib) (38). Immunoblot analyses confirmed selective inhibition of AXL in H4006 cells treated with R428 (Supplemental Figure 2A). Combined treatment with R428 and OSI provided enhanced therapeutic activity compared with R428 or OSI alone, similar to combined MRX-2843 and OSI (Supplemental Figure 2B). In contrast, H4006-OSIR cells were not sensitive to combined R428 and OSI.

### MERTK and MERTK ligands are upregulated in EGFR^MT^ NSCLCs treated with OSI.

**Figure 6:**
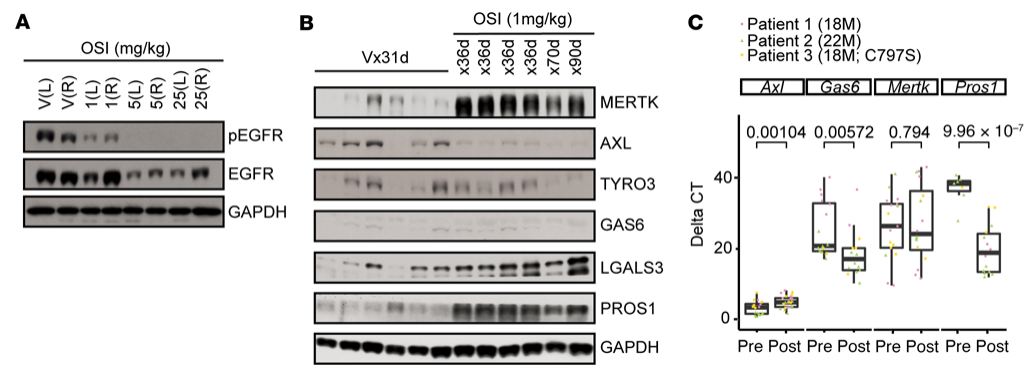
MERTK and/or MERTK ligands are upregulated in *EGFR*^MT^ NSCLCs treated with OSI tumor xenografts and patient samples. (**A** and **B**) 24- to 30-week-old male or female athymic Nude-*Foxn1^nu^* mice with established s.c. H4006 xenograft tumors (200–450 mm^3^) were treated with the indicated dose of OSI or vehicle (V) once daily. (**A**) Treatment was administered for 3 days. Tumors were dissected 3 hours after the last administration of OSI, and phosphorylated and total EGFR were detected by immunoblot. L, left; R, right. (**B**) Treatment was administered for the indicated number of days. Tumors were dissected and TAM receptors and ligands were assessed by immunoblot. (**C**) Three sets of paired pre- and posttreatment tumor biopsies from patients with *EGFR^MT^* NSCLC treated with OSI were obtained, and expression of genes encoding TAM receptors (*AXL* and *MERTK*) and their ligands (*GAS6* and *PROS1*) was determined by quantitative reverse-transcription PCR. Median ΔCt values and standard errors are shown. Significant differences in gene expression after treatment (*P* < 0.05) were determined using LMM. See complete unedited blots in the supplemental material.

To determine the dose of OSI required to inhibit EGFR phosphorylation in H4006 cells in vivo, mice with established H4006 tumors were treated with OSI or vehicle administered once daily for 3 days by oral gavage. EGFR phosphorylation was inhibited in tumors from mice treated with OSI in a dose-dependent manner, with doses as low as 1 mg/kg providing EGFR inhibition (Figure 6A). In a second study, mice with established tumors were treated with vehicle or 1 mg/kg OSI, and expression of TAM receptors and their ligands was evaluated. MERTK protein was dramatically increased in tumors after treatment with OSI for 36 days relative to tumors treated with vehicle, and increased MERTK was evident even after tumors were treated for 90 days (Figure 6B). PROS1 ligand was also robustly upregulated, and expression of Galectin-3 (LGALS3), which is thought to be a MERTK-selective ligand (39), was elevated to a lesser extent. AXL was downregulated and TYRO3 levels were not consistently changed after treatment. Minimal expression of GAS6 was detected.

Similarly, TAM receptor and ligand expression were assessed in 3 paired pre- and posttreatment tumor biopsies from patients with *EGFR^MT^* NSCLC that progressed during treatment with OSI, including one tumor that acquired an *EGFR-C797S* mutation (Supplemental Table 5). Gene-expression levels were determined by quantitative PCR, where ΔCt is the number of PCR cycles required to attain a defined amount of product and ΔΔCt is the difference between median ΔCt values from matched pre- and posttreatment samples, with negative values reflecting increased expression. *PROS1* and *GAS6* were significantly upregulated in samples collected after disease progression during OSI therapy (ΔΔCt = –18.96 and –3.51, respectively) (Figure 6C). *MERTK* expression was not significantly different after OSI treatment, but there was a trend toward increased expression (ΔΔCt = –3.98). In contrast, *AXL* expression was significantly decreased (ΔΔCt = 2.26).

### Treatment with OSI in combination with MRX-2843 provides durable regression of EGFR^MT^ tumors in vivo.

**Figure 7:**
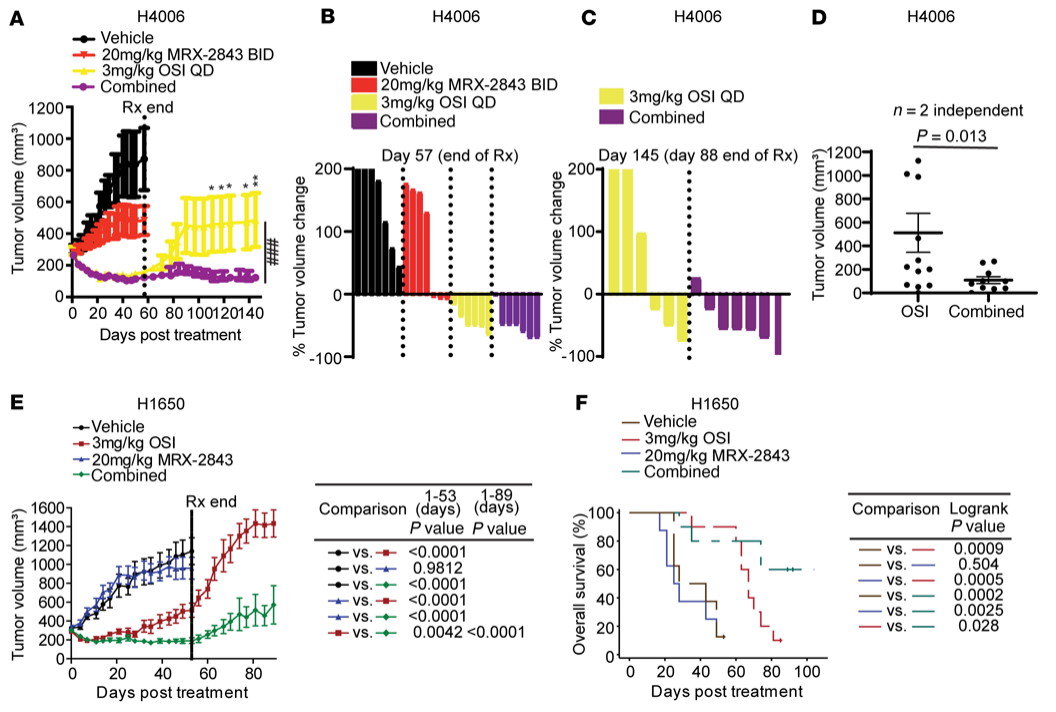
OSI and MRX-2843 combination therapy provides sustained inhibition of *EGFR*^MT^ tumor growth in vivo. Mice with s.c. H4006 tumors (**A**–**D**, 200–450 mm^3^) or H1650 tumors (**E** and **F**, 200–450 mm^3^) were treated with once daily (QD) OSI or twice daily (BID) MRX-2843, OSI and MRX-2843 combined, or vehicle only. Tumors were measured twice weekly during treatment and once weekly after treatment. (**A**) Mean values and standard deviations are shown. *P < 0.05; **P < 0.01; unpaired *t* test 1-way ANOVA; *n* = 6 for 3 mg/kg OSI, n=7 for vehicle and OSI and MRX-2843 combined, and n=8 for 20 mg/kg MRX-2843. ^###^*P* < 0.001, 2-way ANOVA. (**B** and **C**) Waterfall plots showing percentage change in tumor volume at end of treatment (day 57) or 88 days after treatment was stopped (day 145). The *y* axes are truncated at 200%. (**D**) Mean tumor volumes and standard deviations from 2 independent experiments 88 day after treatment was stopped. (**E**) Mean values and standard deviations are shown (*n* = 8–10, LMM). (**F**) Mice with tumor volume greater than 1500 mm^3^ or significant tumor ulceration were removed from study and differences in survival were determined. Plus signs indicate censored mice.

To determine the impact of treatment with MRX-2843 and OSI on *EGFR^MT^* tumor growth, mice with established H4006 xenografts were treated with 3 mg/kg OSI once daily, a subtherapeutic dose of MRX-2843 (20 mg/kg twice daily), MRX-2843 and OSI combined, or vehicle. Treatment with the combination was well tolerated, with mice losing less than 5% of body weight on average and no single mouse with greater than 15% weight loss (Supplemental Figure 3). During treatment, mice receiving OSI alone or with MRX-2843 exhibited similar tumor regression and mice treated with MRX-2843 had reduced tumor volume compared with vehicle-treated mice, with 3 of 7 exhibiting tumor regression (Figure 7, A and B). After 57 days, treatment was stopped and tumor volumes were monitored for an additional 88 days in cohorts treated with OSI monotherapy or OSI and MRX-2843 combination therapy. During this period, half of the tumors treated with OSI alone regrew (Figure 7, A and C). In contrast, tumors that had been treated with the combination therapy did not exhibit significant growth, even after treatment ended (Figure 7A). More specifically, 6 of 7 tumors treated with OSI and MRX-2843 exhibited sustained regression, and minimal growth occurred in the remaining tumor (Figure 7, B and C). Combined data from 2 independent experiments demonstrated a durable and significant reduction in tumor volume in mice treated with the combination therapy compared with mice treated with OSI alone (Figure 7D).

The combination was also more effective in an OSI-refractory model. H1650 cells are relatively resistant to EGFR TKIs, including OSI, compared with other *EGFR^MT^* NSCLC cell lines (31, 40–42). Indeed, OSI monotherapy was not sufficient to prevent disease progression in mice with H1650 tumor xenografts, even during treatment (Figure 7E). In contrast, treatment with MRX-2843 and OSI provided enhanced tumor growth control compared with OSI alone, and this difference became more pronounced after treatment was stopped. Further, treatment with the combination significantly prolonged survival compared with tumors treated with OSI alone (Figure 7F).

## Discussion

OSI is now the preferred front-line treatment for *EGFR^MT^* NSCLC due to its superior efficacy and improved OS compared with that of patients treated with earlier generation EGFR TKIs (18, 19). Unfortunately, only 3% of patients have complete responses to OSI and a majority have residual tumor (18), typically leading to relapse. A deeper understanding of the molecular mechanisms contributing to OSI resistance may provide strategies for improving patient response and survival. In the current study, we provide evidence that MERTK can drive residual tumor survival and/or growth during OSI treatment.

Although EGFR and MERTK were expressed on both *EGFR^MT^* parental and OSIR cells, distinct roles could be demonstrated. In parental cells, EGFR and MERTK could be coimmunoprecipitated and treatment with either OSI or MRX-2843 weakened this interaction, suggesting kinase dependence. Stimulation with EGF or GAS6 enhanced downstream signaling in parental *EGFR^MT^* cells, and EGFR and MERTK shared common signaling pathways that promote tumor cell survival and proliferation, including PI3K/AKT and MAPK/ERK pathways (28). In contrast, EGFR and MERTK did not coimmunoprecipitate from OSIR cell lysates, and only MERTK kinase was active in OSIR cells. EGFR phosphorylation at Y1068 has previously been shown to correlate with downstream activation of PI3K/AKT (8), an important pathway for cell survival. Here, we found that EGFR-Y1068 phosphorylation was not consistently detected and was not responsive to EGF ligand stimulation in OSIR cells, indicating that the EGFR pathway was dysfunctional. In contrast, OSIR cells were still responsive to GAS6 stimulation, leading to enhanced oncogenic signaling, and signaling downstream of GAS6 was blocked by treatment with the MERTK kinase inhibitor MRX-2843, implicating MERTK as a mediator. Indeed, OSIR cells were more responsive to MRX-2843 than OSI-sensitive parental cells, suggesting increased dependence on MERTK.

MRX-2843 is a dual FLT3 and MERTK kinase inhibitor with limited off-target activity (33, 34). Here, we identified MERTK L593 as a gatekeeper residue for MRX-2843. The MERTK-L593G mutant protein was functional and retained kinase activity. Stimulation with GAS6 increased downstream AKT signaling in 633-shMERTK cells expressing the L593G mutant relative to control 633-shMERTK cells and 633-shMERTK cells expressing the MERTK-K619R kinase-dead mutant protein. While treatment with MRX-2843 was sufficient to inhibit the remaining endogenous MERTK and downstream AKT phosphorylation in both 633-shMERTK cells and 633-shMERTK cells expressing the kinase-dead mutant, MRX-2843 did not inhibit MERTK or AKT activity in 633-shMERTK cells expressing the L593G mutant protein. These data provide compelling evidence that MERTK mediates downstream bypass signaling in response to GAS6 stimulation and implicate MERTK inhibition as the mechanism of MRX-2843 therapeutic activity in *EGFR^MT^* cells.

The MERTK pathway was also activated in *EGFR^MT^* tumor xenografts in response to treatment with OSI, consistent with a role for MERTK in OSI resistance in vivo. MERTK and the ligands PROS1 and LGALS3 were upregulated in H4006 tumors treated with OSI, suggesting MERTK activation via autocrine or paracrine mechanisms. Published reports have demonstrated both LGALS3 expression and binding capacity as independent poor prognostic markers in lung cancer patients (43), and treatment with an LGALS3 inhibitor was sufficient to reduce NSCLC tumor growth and metastasis in murine models (44). Similarly, *GAS6* and *PROS1* were significantly increased, and there was a trend toward increased *MERTK* expression in tumor biopsies from patients with *EGFR^MT^* NSCLC after treatment with OSI. While GAS6 can activate both MERTK and AXL, AXL is not activated by PROS1 (45). Thus, these data are consistent with induction of autocrine-mediated MERTK signaling in both murine xenografts and patients treated with OSI.

Expression of AXL, another member of the TAM RTK family, has been previously correlated with resistance to OSI and other EGFR TKIs, and AXL inhibition restored sensitivity to EGFR TKIs in AXL-expressing *EGFR^MT^* NSCLC cells (31, 46, 47). Further, AXL was frequently upregulated in erlotinib-resistant *EGFR^MT^* derivative lines compared with parental cells, and AXL and GAS6 were increased in tumors with acquired erlotinib resistance (46). Forced expression of AXL, but not a kinase-deficient mutant, was sufficient to induce erlotinib resistance in erlotinib-sensitive tumor cells (46). Similarly, we observed increased AXL expression in a subset of OSIR cell-line derivatives. In addition, treatment with either MRX-2843 or R428 sensitized H4006 cells to OSI, while H4006-OSIR cells were resensitized by treatment with MRX-2843, but not R428. These data suggest that in some cases both MERTK and AXL are critical determinants of OSI sensitivity, while in others, one family member can play a selective role, perhaps depending on context or even varying from patient to patient. Further biomarker studies are needed to predict whether inhibitors that specifically target MERTK or AXL or dual-specificity inhibitors will provide the best therapeutic approach, taking into account the potential to apply precision medicine approaches to identifying the best course of action for individual patients and the possibility of increased toxicity associated with less selective inhibitors.

MRX-2843 and OSI mediated potent and synergistic antitumor activity in *EGFR^MT^* NSCLC cell cultures, suggesting that the enhanced therapeutic efficacy and synergistic interactions we observed in response to combined MRX-2843 and OSI were due to target inhibition directly in tumor cells (Figure 5, D–F). However, nude mice do have functional NK cells, and TAM kinases have protumorigenic roles in NK cells (48). Thus, it is possible that some of the therapeutic activity we observed in mice treated with the combination was mediated by MERTK inhibition in NK cells. This is especially important given the known resistance to immune checkpoint blockade in *EGFR^MT^* lung cancers relative to that in NSCLC patients without *EGFR* mutations (49–52) and the possibility that targeting MERTK could overcome this immune resistance. Additional studies are needed to understand the impact of treatment with MRX-2843 and OSI combination therapy on antitumor immunity in *EGFR^MT^* NSCLC models.

A complete response to targeted therapy alone is rare in NSCLC and other tumors. Residual disease provides a reservoir of persisting tumor cells, and resistance to therapy can be clonally derived from this population and/or evolve through adaptive changes in response to TKI therapy (53, 54). A better understanding of the mechanisms underlying the survival of persisting cells is necessary for designing systematic therapeutic strategies. Combined treatment with OSI and the MERTK inhibitor MRX-2843 demonstrated superior inhibition of downstream signaling, cell expansion, and colony formation compared with that in cells treated with either agent alone. Most importantly, *EGFR^MT^* tumors treated with combination therapy exhibited durable tumor regression that persisted even after treatment had ended for more than 12 weeks, while half of tumors treated with OSI alone regrew. In our previous studies, tumors recovered from mice after treatment with the combination therapy consisted primarily of nonproliferative fibrous tissue with low cellularity (28). Collectively, our data demonstrate a role for MERTK as a mediator of oncogenic signaling and driver of residual tumor growth in *EGFR^MT^* NSCLCs treated with OSI and provide strong rationale for treatment of *EGFR^MT^* NSCLC patients with OSI and MRX-2843 combined. Based on these findings, we have initiated a phase I trial to determine the safety and therapeutic benefits of this combination in patients (ClinicalTrials.gov NCT04762199).

## Methods

### Cell culture.

CDK4/hTERT-immortalized HBEC-3KT cells were cultured in airway epithelial cell basal medium supplemented with the Bronchial Epithelial Cell Growth Kit (ATCC). All other cell lines were cultured in RPMI-1640 medium supplemented with 10% fetal bovine serum and penicillin/streptomycin. OSIR cell lines were established by gradually escalating the concentration of OSI from 10 nM to 3 μM until parental cell lines could tolerate to 3 μM OSI treatment in the lab. Parental and OSIR cell line identities were confirmed by short-tandem repeat analysis, and cell lines were confirmed mycoplasma negative. MRX-2843 was synthesized as described previously (33, 34). See Supplemental Tables 1 and 2 for additional information regarding sources of all parental cell lines and reagents.

### EGFR open-reading frame sequencing.

Total RNA was extracted using the QIAGEN RNeasy Mini Kit (QIAGEN) and reverse transcribed with SuperScript III Reverse Transcriptase (Invitrogen). Full-length EGFR was amplified using high-fidelity PfuUltra II Fusion HotStart DNA Polymerase (Agilent Technologies) with the following primers: upstream, GCCAAGGCACGAGTAACAAGC; downstream, GCTTTGTGGCGCGACCCTTAG. The PCR product was purified using the QIAquick PCR Purification Kit (QIAGEN) before sequencing by Eurofins Genomics. Sequencing primers are listed in Supplemental Table 3.

### Cell-expansion assay.

Cells were infected with 0.15 PFU/cell Nuclight Red Lentivirus for 48 hours before selection in 1 μg/ml puromycin as described previously (28). Nuclight Red–expressing cells (3000/96 wells) were cultured overnight and then treated with indicated reagents, and cell numbers were counted at 2-hour intervals over 3 to 5 days using the Incucyte ZOOM system (Essen Bioscience). All Nuclight Red–positive cell lines were established in the lab using parental cell lines.

### Clonogenic assay.

Cells (1200/12 wells) were treated with indicated reagents for 7 to 10 days before colonies were stained with crystal violet (0.2% w/v in 25% methanol) and counted using a GelCount Colony b Counter (Oxford Optronix).

### Preparation of GAS6 conditioned medium.

HEK-293TN cells stably expressing GAS6 were provided by Raymond Birge (Rutgers University, Newark, New Jersey, USA), and medium containing GAS6 was prepared as described (55). Briefly, cells at approximately 80% confluency were serum starved in DMEM medium overnight and then cultured in serum-free DMEM containing 10 μg/ml vitamin K1 (Hospira) for 72 hours. The conditioned medium was collected, filtered through a 0.22 μm filter, and stored at 4°C until further use.

### Cell signaling.

Cells were starved overnight before treatment with EGF, recombinant GAS6, GAS6-conditioned medium, or recombinant PROS1 for 10 minutes. Where indicated, cultures were treated with OSI and/or MRX-2843 in serum-free DMEM for 2 hours before ligand stimulation. Cell lysates were prepared and assessed by immunoblot analysis. Phosphorylated and total MERTK were detected after pervanadate treatment and immunoprecipitation using MERTK antibody (34). MERTK antibodies or EGFR antibodies were used for coimmunoprecipitation. HRP-conjugated secondary antibodies were used for detection of proteins using enhanced chemiluminescence. Antibodies are indicated in Supplemental Table 4. Immunoblot images shown are representative of at least 3 independent experiments.

### shRNA/siRNA knockdown.

Lentiviral particles were used to introduce shRNA targeting human MERTK in the 3′ UTR (Oligo ID: TRCN0000000862, Open Biosystems) into 633 cells as previously described and selected with 1.5 μg/ml puromycin (56). Single-cell clones were derived by limiting dilution and maintained with selection. Cells were transfected with 100 nM EGFR siRNA, AXL siRNA, MERTK siRNA, or nontargeting siRNA for 24 hours before cell expansion, colony formation, or cell migration assays. siRNAs are described in Supplemental Table 2.

### MERTK mutants.

The predicted gatekeeper mutation (L593G) of *MERTK* in pLNCX2 plasmid was constructed as previously described for the kinase-dead *MERTK* mutant (K619R) (37). Constructs were sequence verified. 633-shMERTK cells were infected with lentiviral particles derived from pLNCX2-*MERTK*, pLNCX2-*MERTK*-K619R, pLNCX2-*MERTK*-L593G, or pLNCX2-*GFP* plasmids and selected with 400 μg/ml geneticin and 1.5 μg/ml puromycin. Single-cell clones were derived by limiting dilution and maintained with selection.

### Incucyte ZOOM cell migration assay.

1000 Cells were cultured in 60 μl RPMI+10% serum in the insert of an IncuCyte ClearView 96-well cell migration plate (Essen Bioscience) for 15 minutes; then the insert was loaded into an Incucyte ClearView reservoir plate containing 200 μl RPMI+10% serum. Migrated cells were counted at 2-hour intervals using the Incucyte ZOOM system with Chemotaxis software (Catalog 9600-0015).

### 3D structures of ligand protein complexes.

The 3D structure of MERTK kinase domain in complex with ADP was retrieved from the Protein Databank (PDB 3BRB). The 3D structure of MERTK in complex with MRX-2843 was obtained by docking the inhibitor into MERTK crystal structure (PDB 4M3Q). The Glide module (57) in standard precision mode with default settings within the Maestro modeling suite (release 2016-2; Schrödinger, LLC) was used as a docking tool. Protein Preparation Wizard, available through Maestro (release 2018-4; Schrödinger, LLC), was used to prepare the complexes for MD simulations. In addition to the default settings, missing side chains and missing loops were added using Prime. To avoid unnatural interatomic clashes, restrained minimization with heavy atom convergence at RMSD 0.3 Å was performed.

### MD simulations.

All MD simulations were performed in the Gromacs 2018.2 simulation package using the CHARMM22 protein force field (58). 3D ligand-protein complexes described in the previous section were used as starting structures for MD simulations. The force-field parameters for all ligands were generated using SwissParam (59). Ligand-protein complexes were minimized in vacuum using the steepest decent algorithm for 5000 steps or until the maximum force of 1000 kJ × mol^–1^ × nm^–1^ was reached. The molecular systems were then solvated in TIP3P water (60), counterions were added for system neutrality, and NaCl was added by replacing water molecules to mimic 0.15M physiological conditions. Solvent minimization was then performed, followed by a 2-step equilibration, during which all heavy atoms of the system, excluding those of water and counterions, were restrained: 0.1 ns in NVT ensemble using the modified Berendsen thermostat (61) set at constant 300 K, and 1 ns in NPT ensemble at constant 1 atm and 300 K using Parinello-Rahman pressure coupling (62). All simulations were conducted using the Leapfrog integrator in periodic boundary conditions. The 12-6 Lennard-Jones potential was used to describe the vdW interactions, and the nonbonded cutoff distance was set at 0.1 nm. Long-range electrostatic interactions were calculated using the particle mesh Ewald method (63). Bonds involving hydrogen atoms were constrained using the linear constraint solver algorithm (LINCS) (64). Production simulations were conducted in the NVT ensemble with all atoms free to move. For each of the 2 ligand-protein complexes, 3 MD trajectories (1 second each) were collected. MDTraj was used to convert MD structures into PDB format. Pipeline Pilot (www.3dsbiovia.com) was used to process the MD structures and calculate the numbers of close contacts between ligands and residues of interest. Molecular visualization and generation of graphics were performed using PyMOL.

### Expression of TAM receptors and ligands in patient samples.

RNA was extracted using the RecoverALL Total Nucleic Acid Isolation Kit (Ambion) according to the manufacturer’s instructions. cDNA was synthesized using the SuperScript III system (Thermo Fisher Scientific). *MERTK* (25), *AXL* (25), *PROS*1 (65), *GAS6* (65), and *RNA18S* (25) transcripts were assessed by quantitative PCR using FAST SYBR Green Master Mix (Thermo Fisher Scientific) and previously described primers. ΔCt was calculated as the number of cycles required to attain a specified or threshold amount of product (Ct) for the gene of interest normalized to the Ct for the 18S rRNA reference gene.

### Xenograft models.

Commercially available athymic Nude-*Foxn1^nu^* mice (Jackson Laboratory) from our colony were bred until the F2 generation, and then new breeders were obtained from the Jackson Laboratory. Cells (5.5 million/mouse) were injected s.c. into the flanks of 24- to 30-week-old male or female athymic Nude-*Foxn1^nu^* mice in 100 μl PBS containing 50% Matrigel (Corning). Tumors were measured twice weekly with calipers, and volume was defined as π*a*^2^*b*/6, where *a* is the shortest diameter measured perpendicular to the longest diameter, *b*. When tumors reached 200 to 450 mm^3^, mice were randomized to groups with statistically similar starting tumor volumes and treated with vehicle (1% polysorbate 80) (16), MRX-2843, OSI (Utanpharma), or a combination. Treatments were administered by oral gavage, and the combination therapy was administered as a single bolus. Mice with obvious tumor ulceration or tumor volume greater than 1800 mm^3^ were removed from study.

### Statistics.

To determine differences in gene expression in patient samples, a linear mixed model (LMM) (66), which is widely used to account for repeated measurements of the same unit (67–69), was fitted separately for each gene. In this model, the outcome is ΔCt, the covariate is treatment condition (pre versus post), and the random intercept is assigned for each patient. Outliers were identified by Cook’s distance (70, 71) with cutoff as 4/*N*, where *N* is the total number of measurements. Reported results were derived by applying LMM on data with outliers removed. LMM analysis was performed on the R platform with package lmer4 (72).

For xenograft studies, tumor volume data sets with 30% or less of measurements collected were censored and final tumor volumes were carried until the end of the study for mice that were removed prematurely due to tumor ulceration or tumor volume. Significant differences in tumor volume were determined by 1- or 2-way ANOVA with Tukey’s or Bonferroni’s post test or using an LMM. The Kaplan-Meier plot for OS was generated and compared between groups using the log-rank test. Univariate analysis between treatment groups and OS were also assessed using Cox’s proportional hazards model.

### Study approval.

Written, informed consent was received for all participants. Formalin-fixed, paraffin-embedded sections from deidentified tumor biopsies were provided by the Emory University Lung SPORE Pathology Core and the Winship Cancer Institute Tissue Procurement and Pathology Core. All animal studies were approved by the Emory University Animal Care and Use Committee.

## Author contributions

DY conceived the study, designed, performed, analyzed, and interpreted experiments, and wrote the manuscript. DKG and DD conceived the study, analyzed and interpreted experiments, and edited the manuscript. JMH performed and analyzed experiments. DK designed, performed, analyzed, and interpreted computational modeling. ZT, LC, and SG analyzed experiments. MB and FS provided patient samples and related clinical data. HSE, XW, and SVF provided suggestions for experiments and edited the manuscript. SSR and TO provided suggestions for experiments and clinical perspective and edited the manuscript. All authors read and agreed with the manuscript.

## Supplementary Material

Supplemental data

## Version 1 06/16/2022

In-Press Preview

## Version 2 08/01/2022

Electronic publication
