# Supplementary material for: MERTK activation drives osimertinib resistance in EGFR-mutant non–small cell lung cancer
Source: J Clin Invest. 2022 Aug 1;132(15):e150517. doi: 10.1172/JCI150517 (PMC9337831; doi:10.1172/JCI150517)

Supplementary Information

MERTK activation drives *EGFR*-mutated non-small cell lung cancer resistant to osimertinib

Dan Yan[1], Justus M. Huelse[1], Dmitri Kireev[2], Zikang Tan[1], Luxiao Chen[3], Subir Goyal[3], Xiaodong Wang[2], Stephen V. Frye[2,4], Madhusmita Behera[3], Frank Schneider[5], Suresh S. Ramalingam[6,7], Taofeek Owonikoko[6,7], H. Shelton Earp[4,8], Deborah DeRyckere[1,*], and †Douglas K. Graham[1,*]

[1]Aflac Cancer and Blood Disorders Center, Children's Healthcare of Atlanta and Department of Pediatrics, Emory University School of Medicine, Atlanta, GA, 30322, USA

[2]Center for Integrative Chemical Biology and Drug Discovery, Division of Chemical Biology and Medicinal Chemistry, Eshelman School of Pharmacy, University of North Carolina at Chapel Hill, Chapel Hill, NC, 27599, USA

[3]Biostatistics and Bioinformatics Shared Resources, Winship Cancer Institute, Emory University School of Medicine, Atlanta, GA 30322, USA

[4]Department of Medicine, UNC Lineberger Comprehensive Cancer Center, Chapel Hill, NC, USA

[5]Department of Pathology, Emory University School of Medicine, Atlanta, GA 30322, USA [6]Winship Cancer Institute, Emory University School of Medicine, Atlanta, GA, 30322, USA [7]Department of Hematology and Medical Oncology, Emory University School of Medicine, Atlanta, GA, 30322, USA

[8]Department of Pharmacology, School of Medicine, University of North Carolina at Chapel Hill, Chapel Hill, NC, 27599, USA

* These authors contributed equally to this work.

Patent numbers to XW and SVF describing MRX-2843 are listed here: PCT/US2012/058298, WO2013052417 A1, US2013/641729, US 9273056, ZL2012800568254, Japanese patent number 6316925, Australian patent number 2012318896, European patent number FR2763988, RU 2631655, HK1201256, US2015/790700, US10179133, KR 10-2063098, PCT/US2015/014573, US 2016/0151372, US 9795606 B2, PCT/US2015/024381, WO2015157128A1, US9555031, US10004755, PCT/US2020/026167, WO2020205967, US2022/0162214, and JPA 2022522539-000000 (to University of North Carolina).

Table S1 Cell line source information.

Cell Line	Source
**HBEC-3KT**	American Type Culture Collection, Manassas, VA
**A549**	
**H226**	
**H2009**	
**PC9**	Immuno-Biological Laboratories Co, Japan
**COLO699**	German Collection of Microorganisms and Cell Culture, Germany
**H2228**	Drs. John Minna and Adi Gazdar, University of Texas Southwestern Medical Center, Dallas, TX
**H4006**	
**H4011**	
**H1975**	
**H1650**	

Table S2 Reagent information.

Reagents	Company	Catalog#
**Recombinant GAS6 (rGAS6)**	R&D Systems	885-GSB
**Recombinant human EGF**		236-EG-200
**Recombinant PROS1 (rPROS1)**	Sino Biological	12179-H08H
**geneticin**	ThermoFisher Scientific	10131027
**EGFR siRNA (EGFRsi)**		EGFRHSS103114
**MERTK siRNA (MERTKsi)**		MERTKHSS116030
**AXL siRNA (AXLsi)**		AXLHSS100897
**Non-targeting siRNA (Vsi)**	Santa Cruz	sc-37007
**Nuclight^{TM}Red lentivirus**	Essen Bioscience	4476
**Puromycin**	TaKaRa	631306

Table S3 Primers used for *EGFR* gene sequencing.

Sequence (5`-3`)
GCCAAGGCACGAGTAACAAGC
TGCCCTGTGCAACGTGGAGA
GTGGTGACAGATCACGGCTC
GACCAAGCAACATGGTCAG
CTGCCTCAGGCCATGAACATC
GAAGCGCACGCTGCGGAGGC
TGAACTACTTGGAGGACCGTC
CAGATAGTCGCCCAAAGTTC
GACGACACCTTCCTCCCAG

Table S4 Antibody information.

Antibody	Catlog #	Clone #	Company	Application
**pAKT**	9271S	polyclonal	Cell signaling	Primary antibodies for immunoblot
**AKT**	9272S	polyclonal		
**pERK**	4376S	20G11		
**ERK**	9102S	polyclonal		
**pEGFR**	2234S	polyclonal		
**EGFR**	2232S	polyclonal		
**pS6**	4858S	D57.2.2E		
**GFP**	2555S	Polyclonal		
**TYRO3**	5585S	D38C6		
**GAPDH**	2118S	14C10		
**TUBULIN**	2125S	11H10		
**AXL**	AF154	Polyclonal	R&D Systems	
**GAS6**	AF885	Polyclonal		
**MERTK**	ab52968	Y323	Abcam	
**LGALS3**	ab2785	A3A12		
**pMERTK**	p186-749	Polyclonal	Phosphosolutions (1)	
**PROS1**	A0384	Polyclonal	Dako	
**MERTK**	MAB8912	125518	R&D Systems	Immunoprecipitation
**EGFR**	MAB1095	102618		
**Goat anti-mouse**	170-6516	Polyclonal	Bio-Rad	Secondary antibodies for immunoblot
**Goat anti-rabbit**	170-6515	Polyclonal		
**Donkey anti-goat**	sc-2020	polyclonal	Santa Cruz Biotechnology	

Table S5 *EGFR* mutation status and EGFR TKI treatment history for patient samples.

	Pre or post	Prior EGFR TKI Y/N	Relapsed on prior EGFR TKI Y/N	Duration of OSI	*EGFR* mutation status	Mechanism of OSI resistance
**Patient 1**	pre	Yes	No	18 months	*EGFR* L858R	
	post					Not known
**Patient 2**	pre	Yes	No	22 months	*EGFR* exon 19 del	
	post					Not known
**Patient 3**	pre	Yes	No	18 months	*EGFR* exon 19	
	post					de novo *EGFR*-C797S mutation

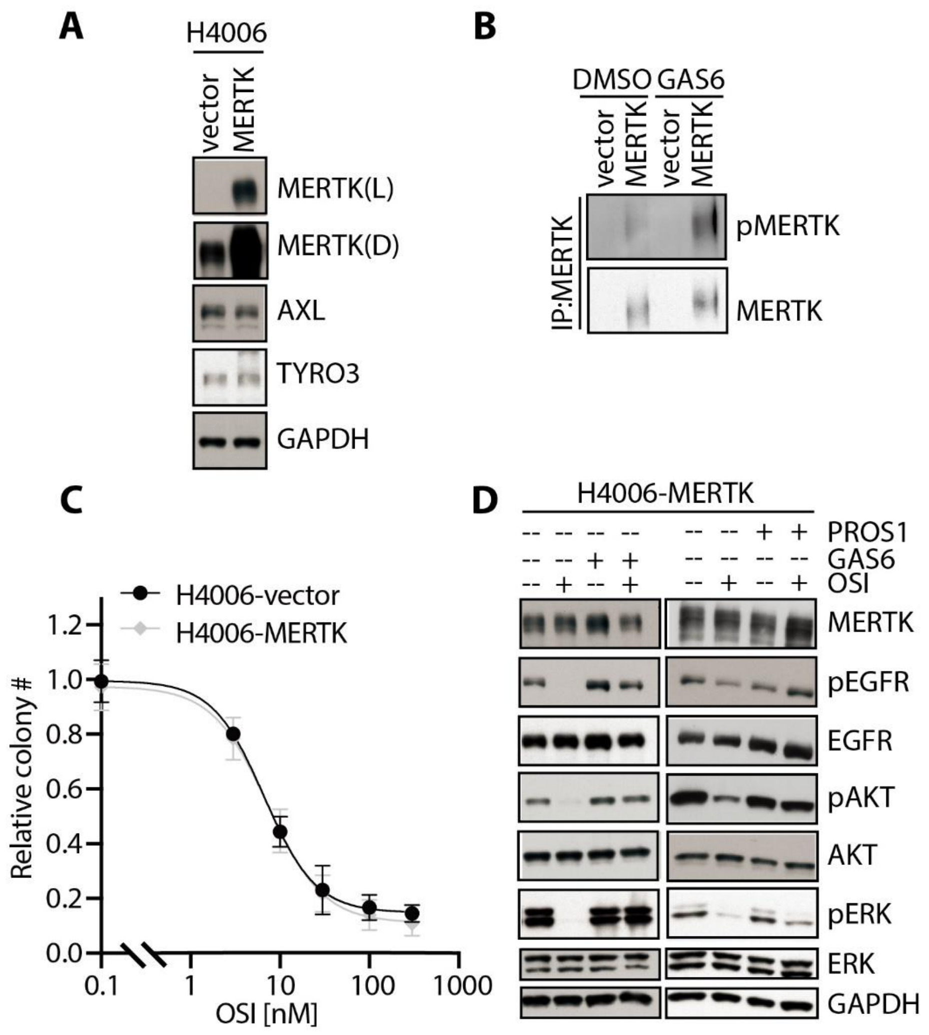

Figure S1 MERTK overexpression is not sufficient to confer OSI resistance. A) H4006 cell lines with MERTK overexpression (MERTK) or empty vector (vector) were established, and expression of TAM receptors was assessed by immunoblot. **B**) H4006-MERTK and H4006-vector cells were serum-starved overnight, then stimulated with or without 50nM GAS6 for 10min and treated with pervanadate and cell lysates were analyzed by immunoblot. **C**) H4006-MERTK and H4006-vector cells were cultured at low density and treated with the indicated concentrations of OSI or vehicle for 8d before cells were fixed and colonies were stained and counted. Mean colony numbers relative to vehicle-treated control cultures and standard deviations from three independent experiments are shown. **D**) H4006-MERTK cells were serum-starved overnight and then treated with DMSO or 100nM OSI for 2h followed by 10min stimulation with or without 50nM GAS6 or 50nM PROS1. Cell lysates were analyzed by immunoblot.

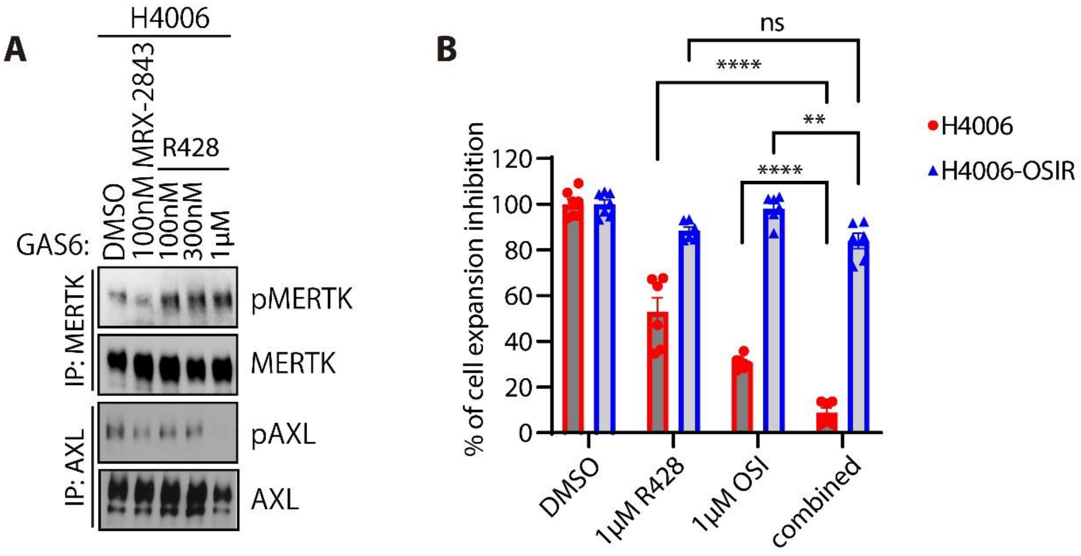

Figure S2 AXL inhibitor R428 sensitizes *EGFR*^{MT} H4006 parental cells to OSI but does not enhance therapeutic efficacy in combination with OSI in OSI resistant (OSIR) H4006 cells. **A**) H4006 cells were serum-starved overnight and then treated with the indicated concentrations of MRX-2843 or R428 for 2h, followed by GAS6 stimulation for 10min. Phosphorylated (denoted by p) and total proteins were detected after pervanadate treatment and immunoprecipitation. **B**) H4006 and H4006-OSIR cells were treated with R428 or OSI alone or combined for 120h and cell numbers were determined using CellTiter Glo reagent. (**, $p<0.01$; ****, $p<0.0001$; ns=not significant; one-way ANOVA).

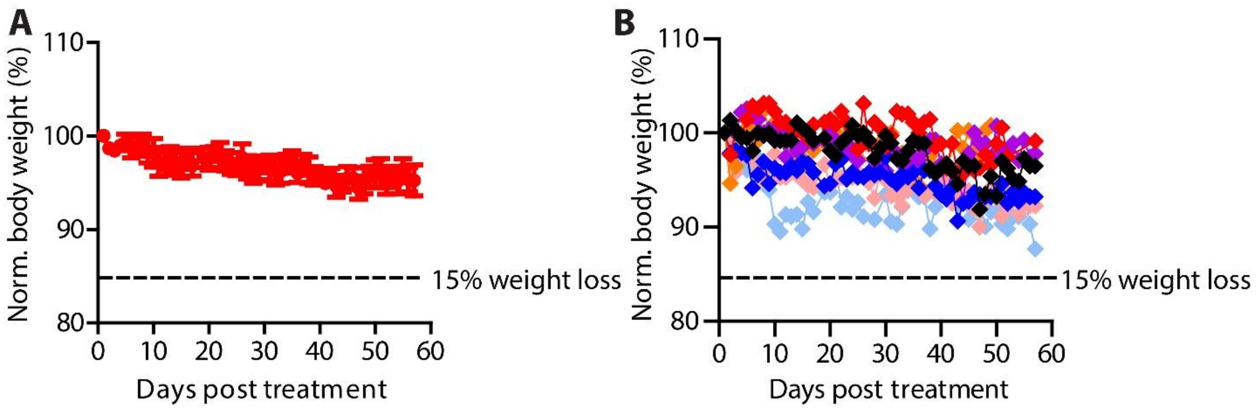

Figure S3 Body weights during treatment with combined OSI and MRX-2843 in mice with H4006 tumor xenografts. A) Mean body weights and standard errors (n=7). **B**) Body weights in individual mice.

1. Zhang W, DeRyckere D, Hunter D, Liu J, Stashko MA, Minson KA, et al. UNC2025, a potent and orally bioavailable MER/FLT3 dual inhibitor. *J Med Chem.* 2014;57(16):7031-41.

Full unedited gel for Figure 1B

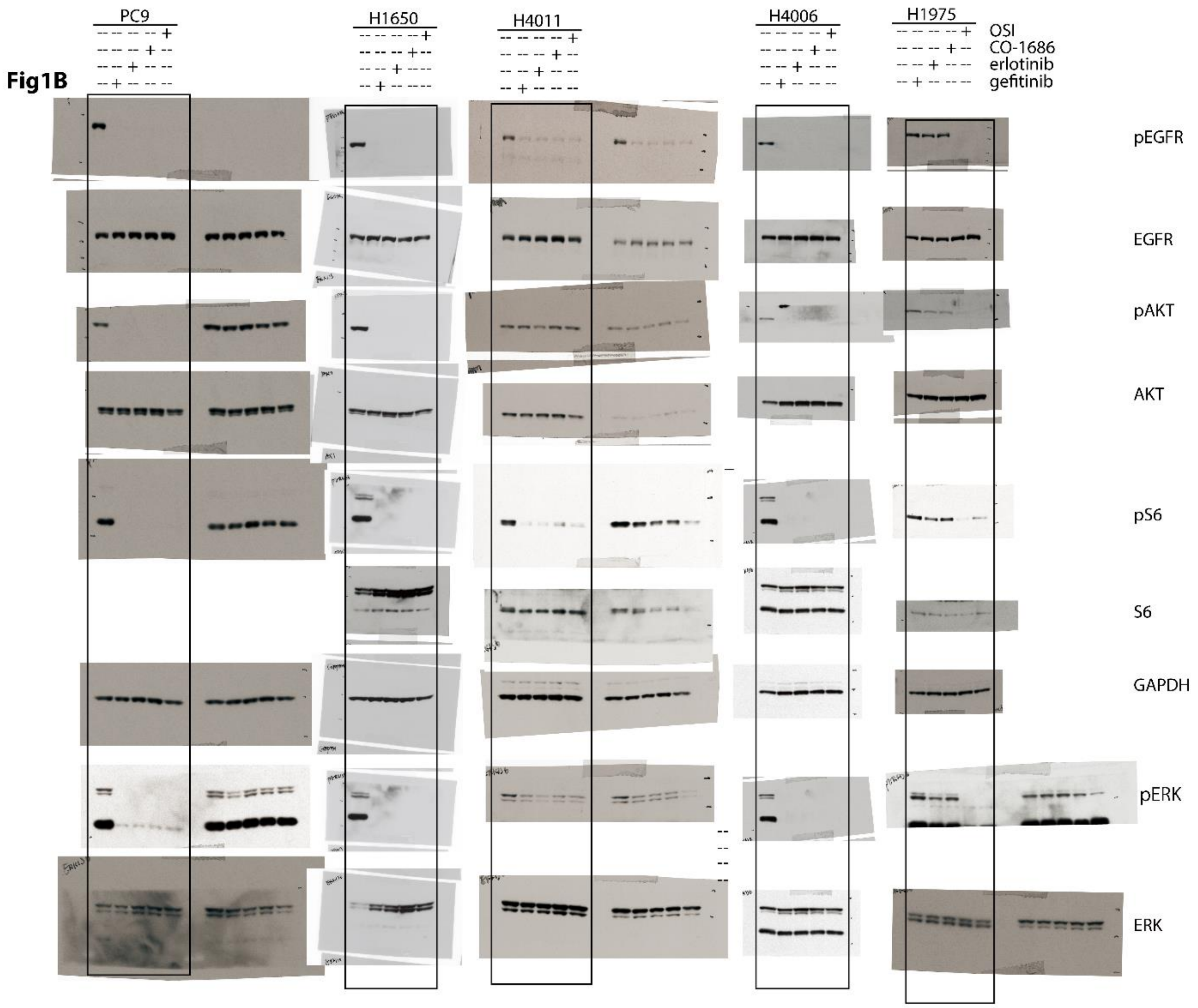

Full unedited gel for Figure 1D

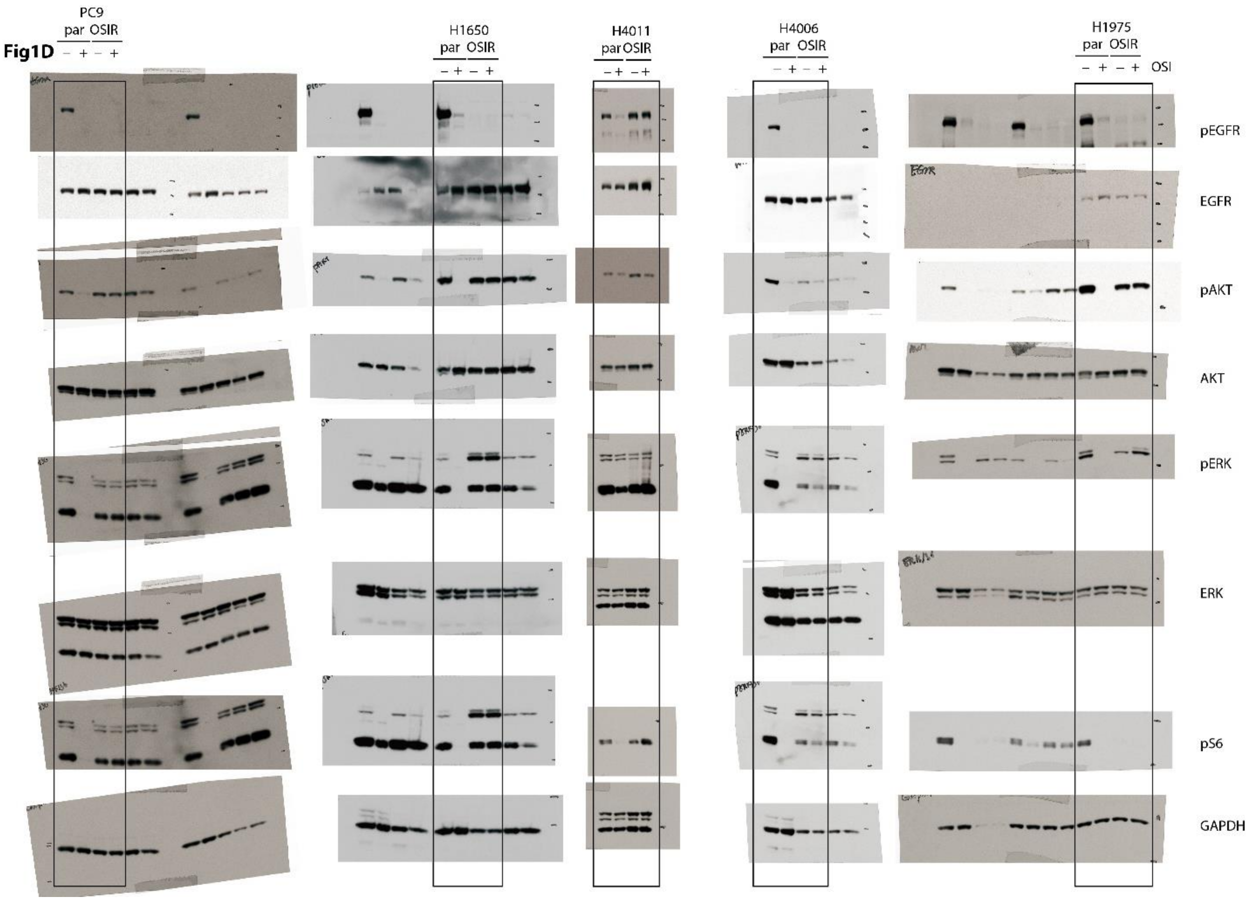

Full unedited gel for Figure 1F

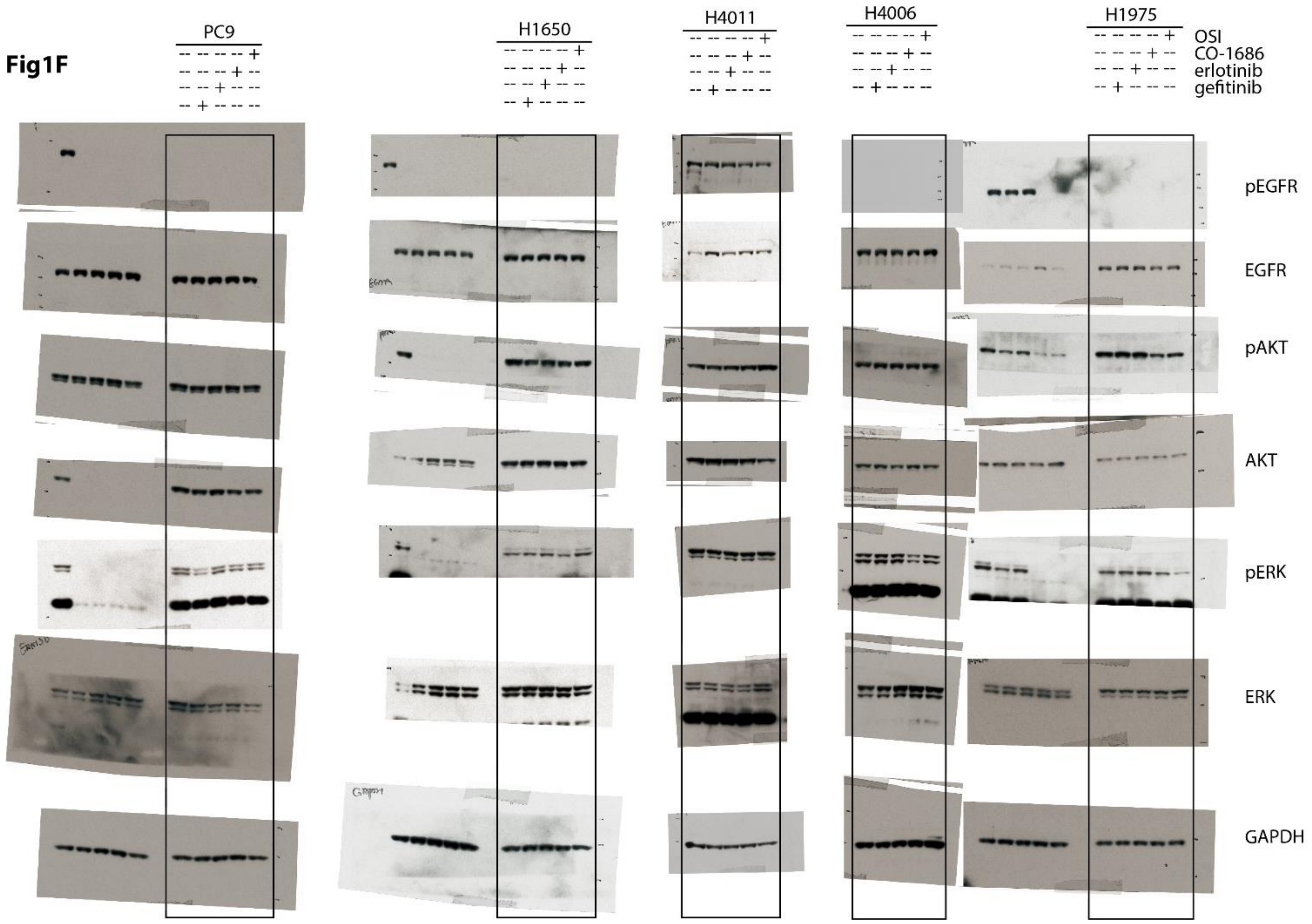

Full unedited gel for Figure 2

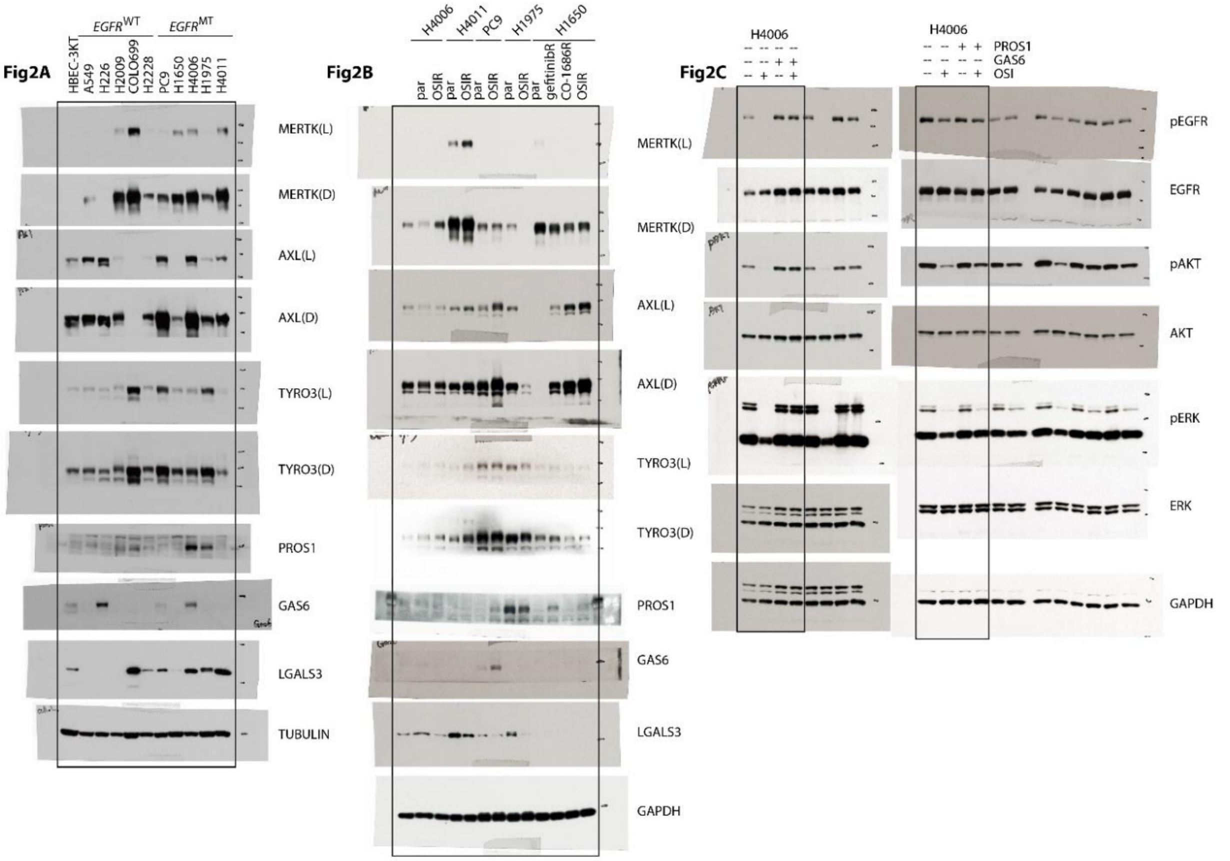

Full unedited gel for Figure 3A and 3B

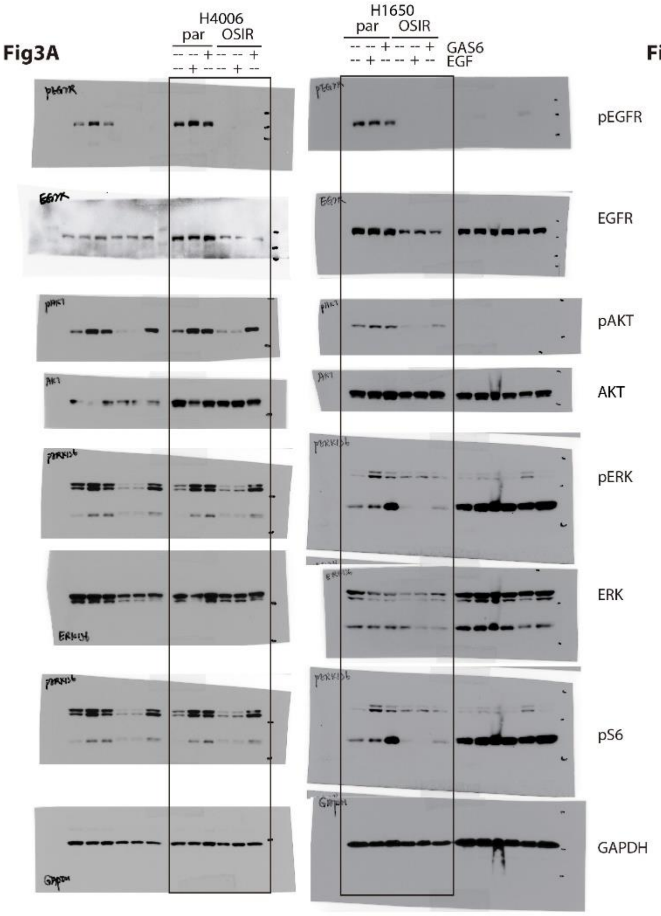

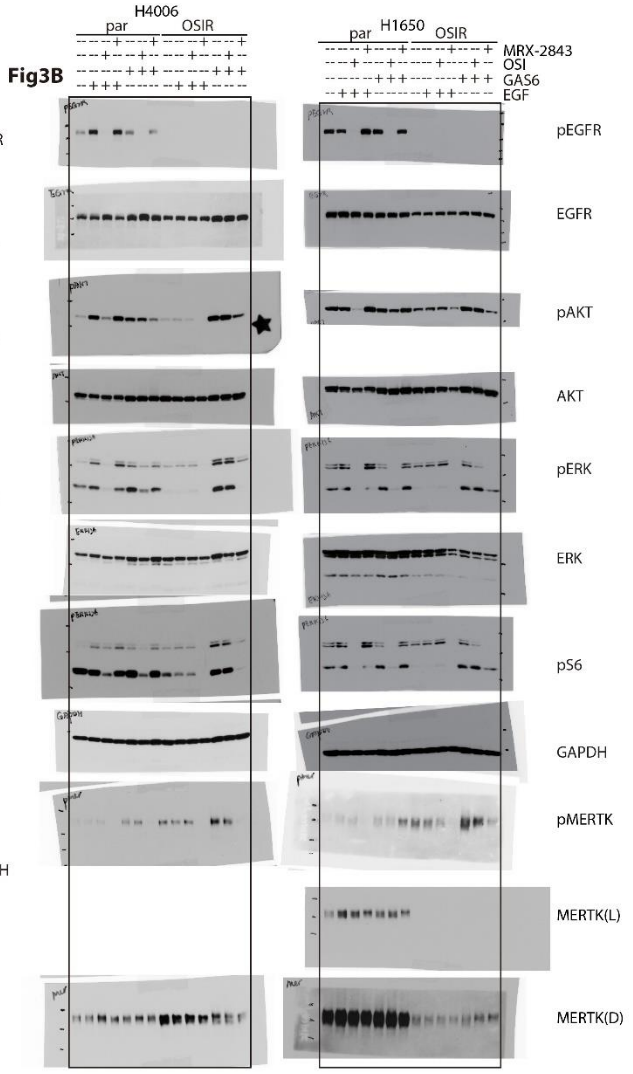

Full unedited gel for Figure 3E, 3I and 3J

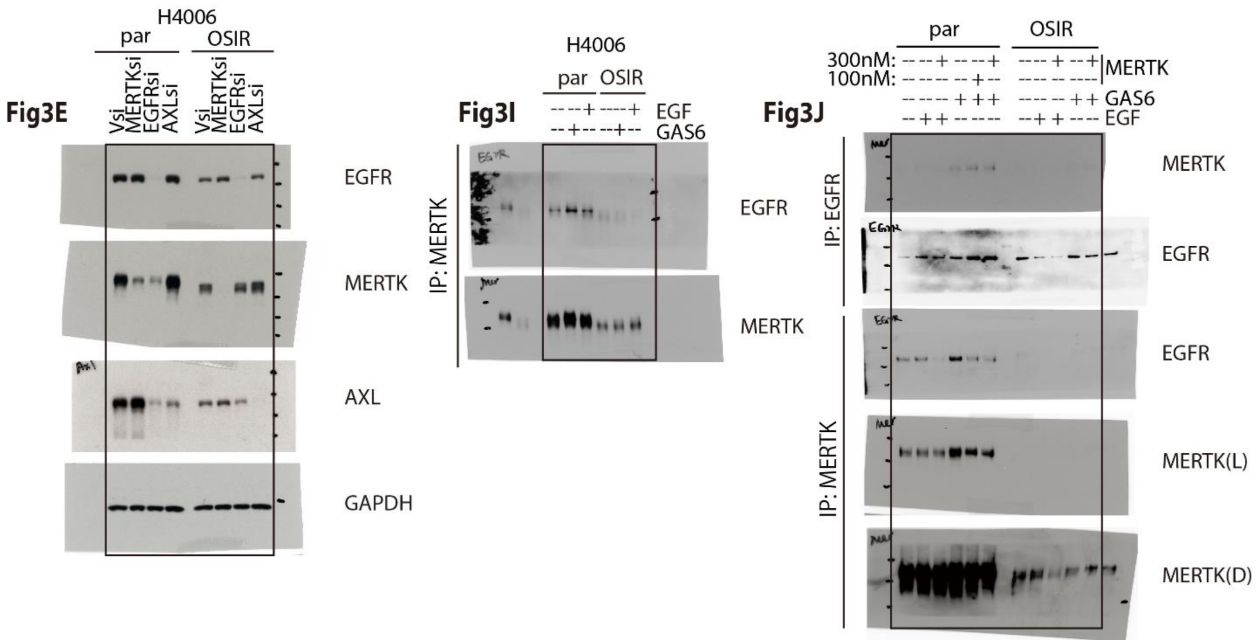

Full unedited gel for Figure 4

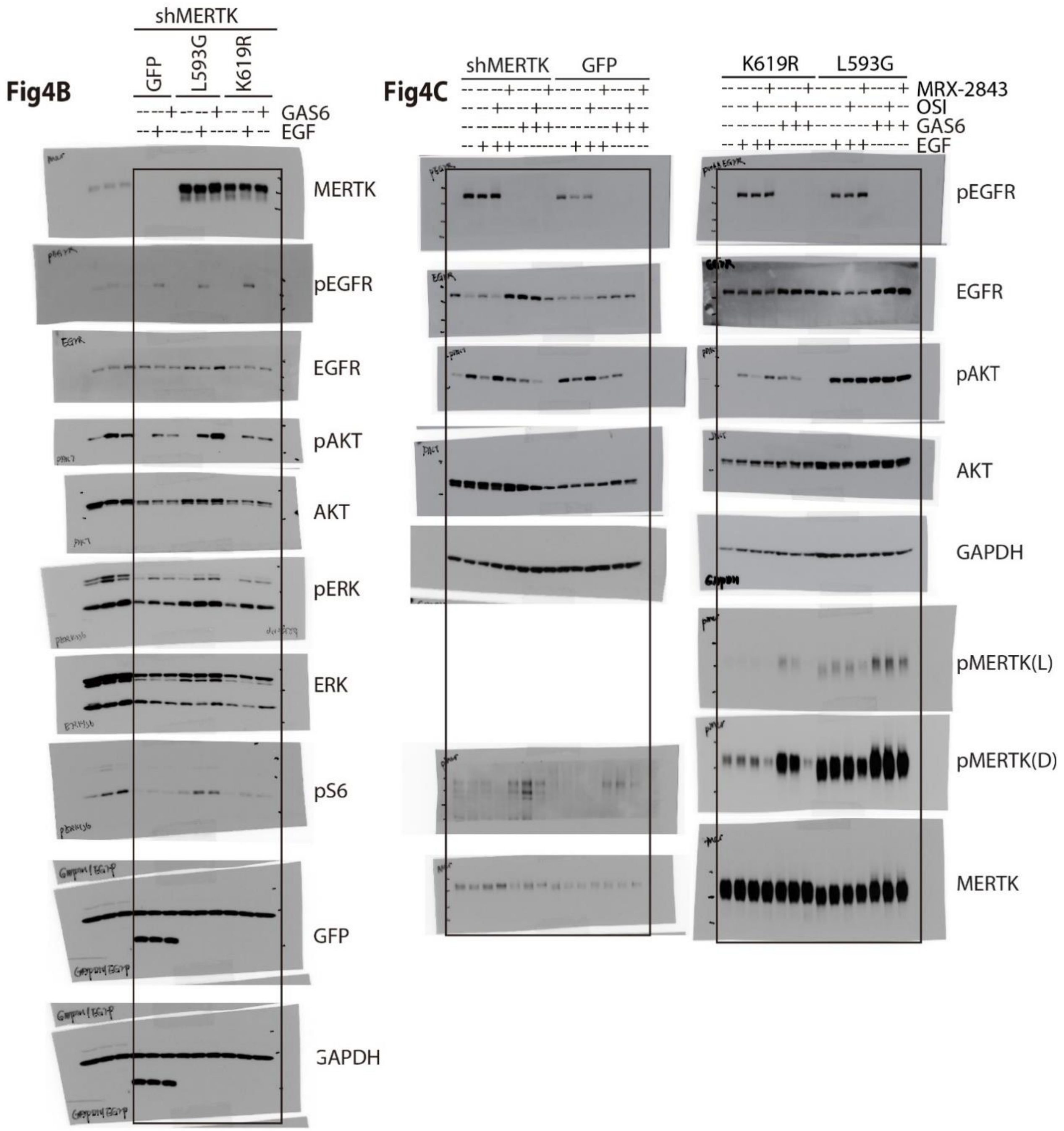

Full unedited gel for Figure 5

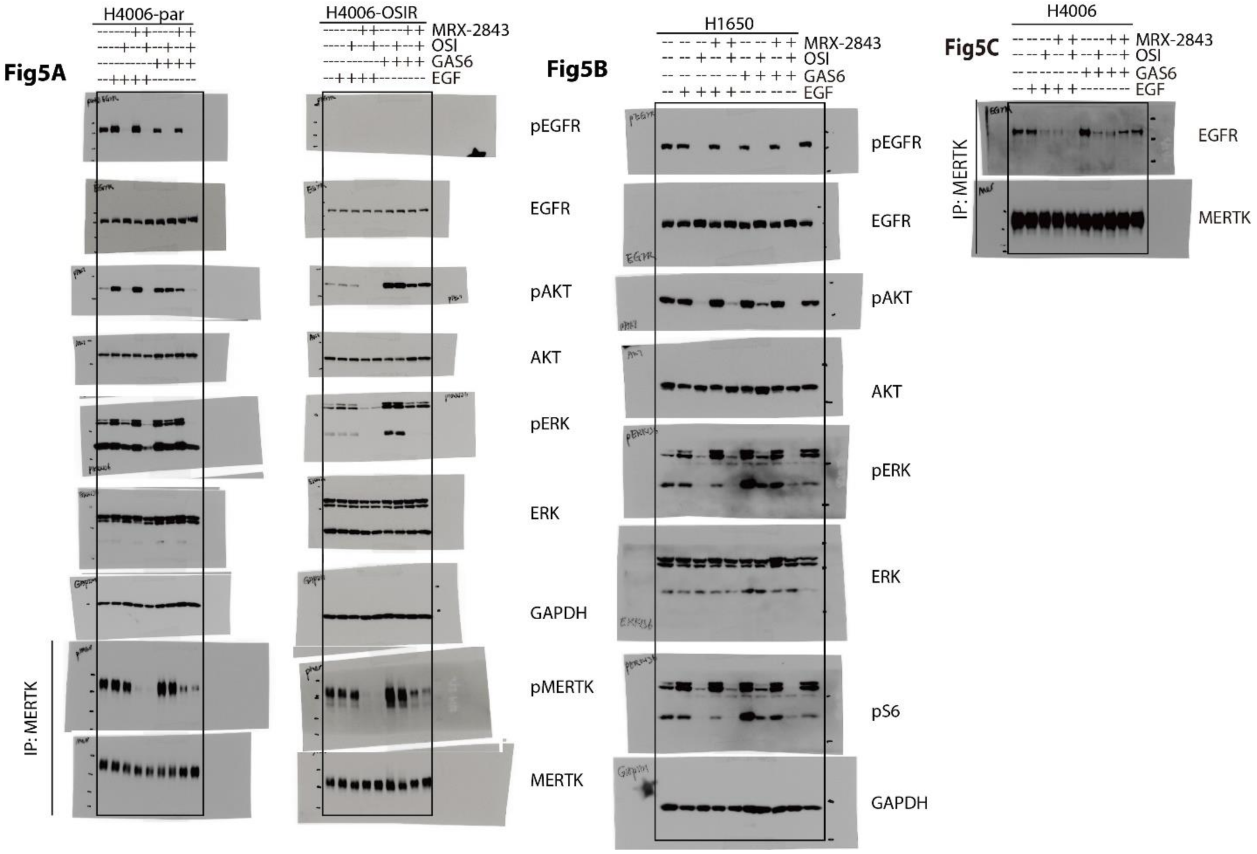

Full unedited gel for Figure 6

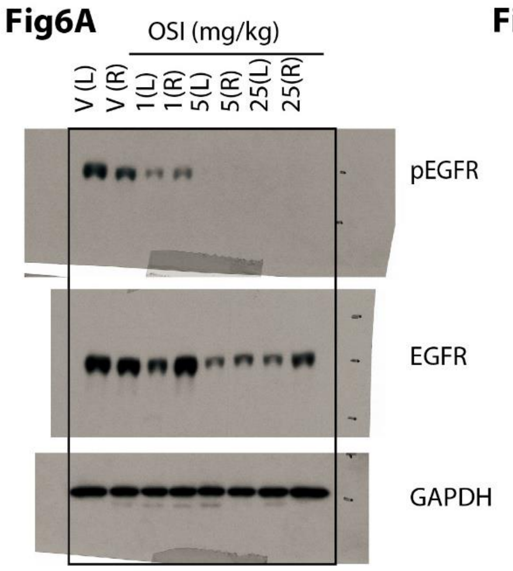

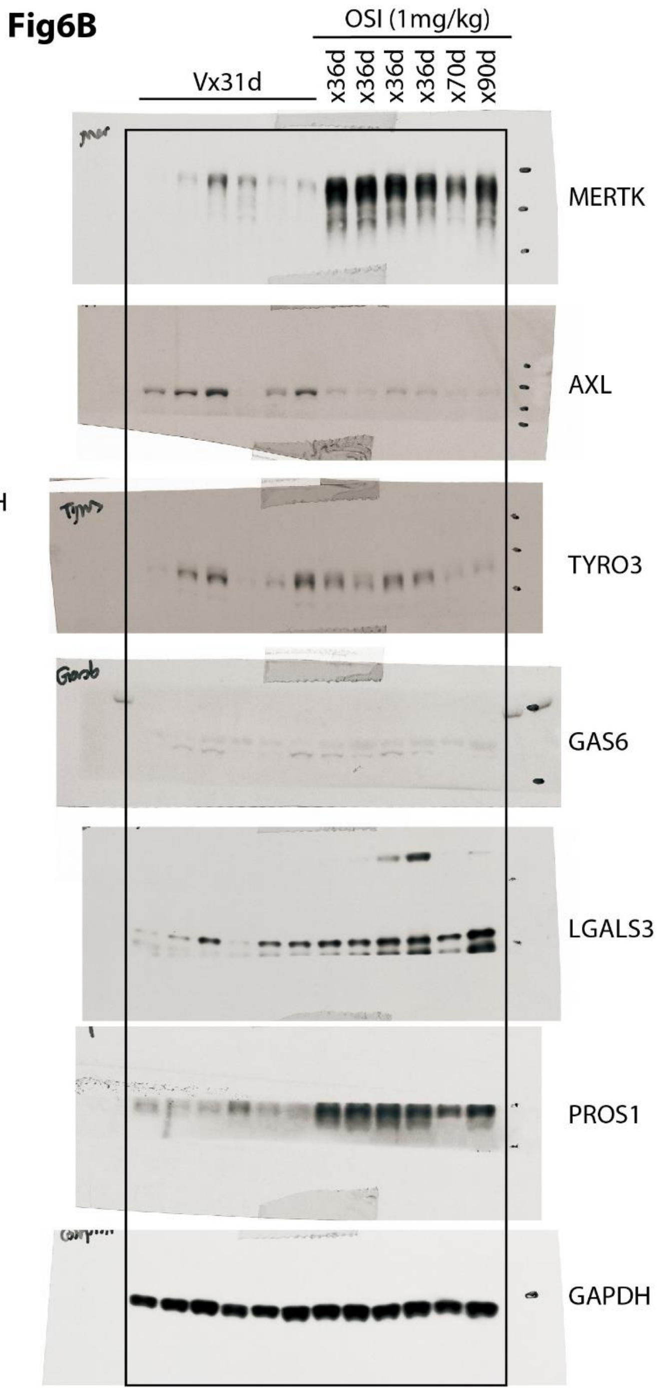

Full unedited gel for Figure S1

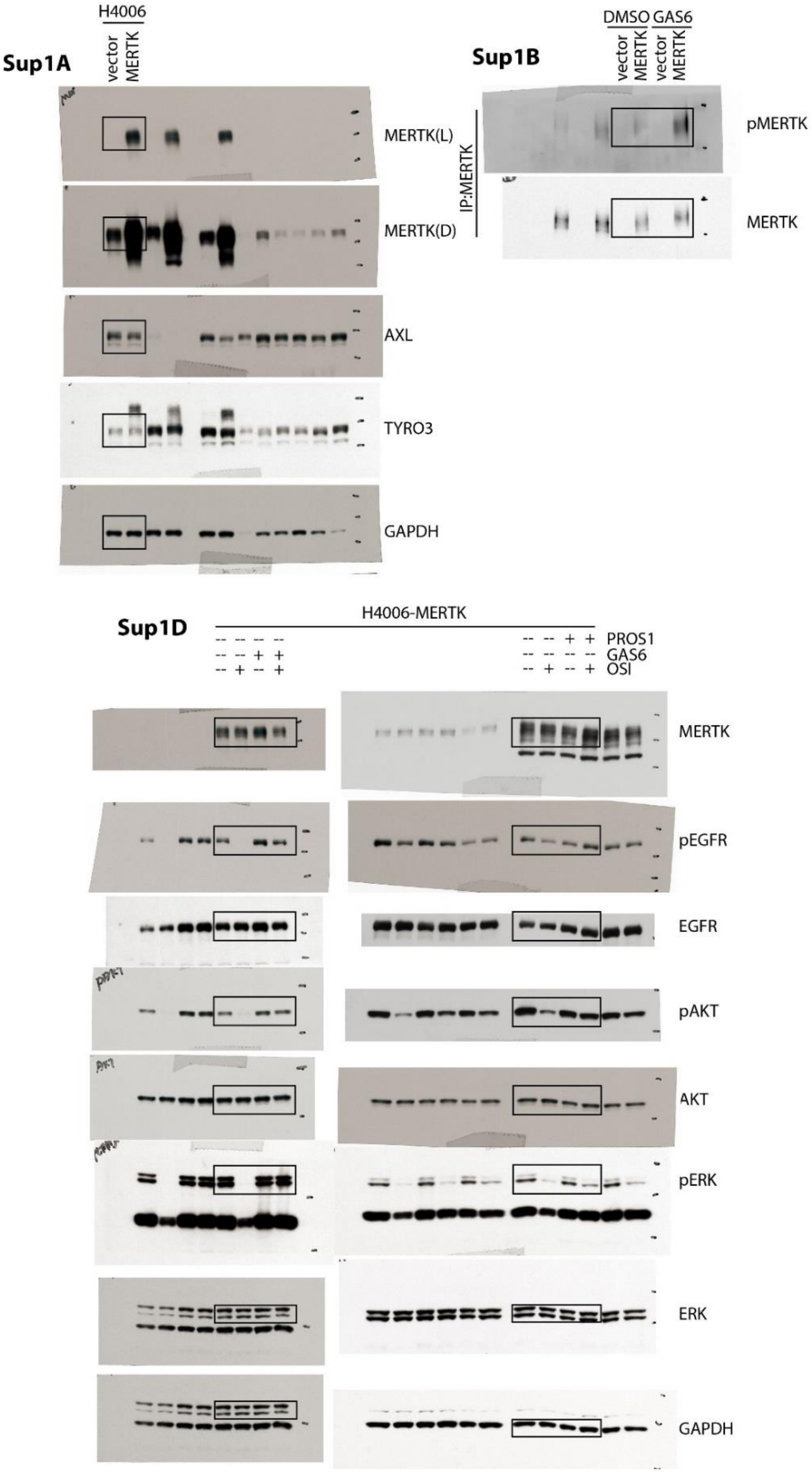

Full unedited gel for Figure S2A

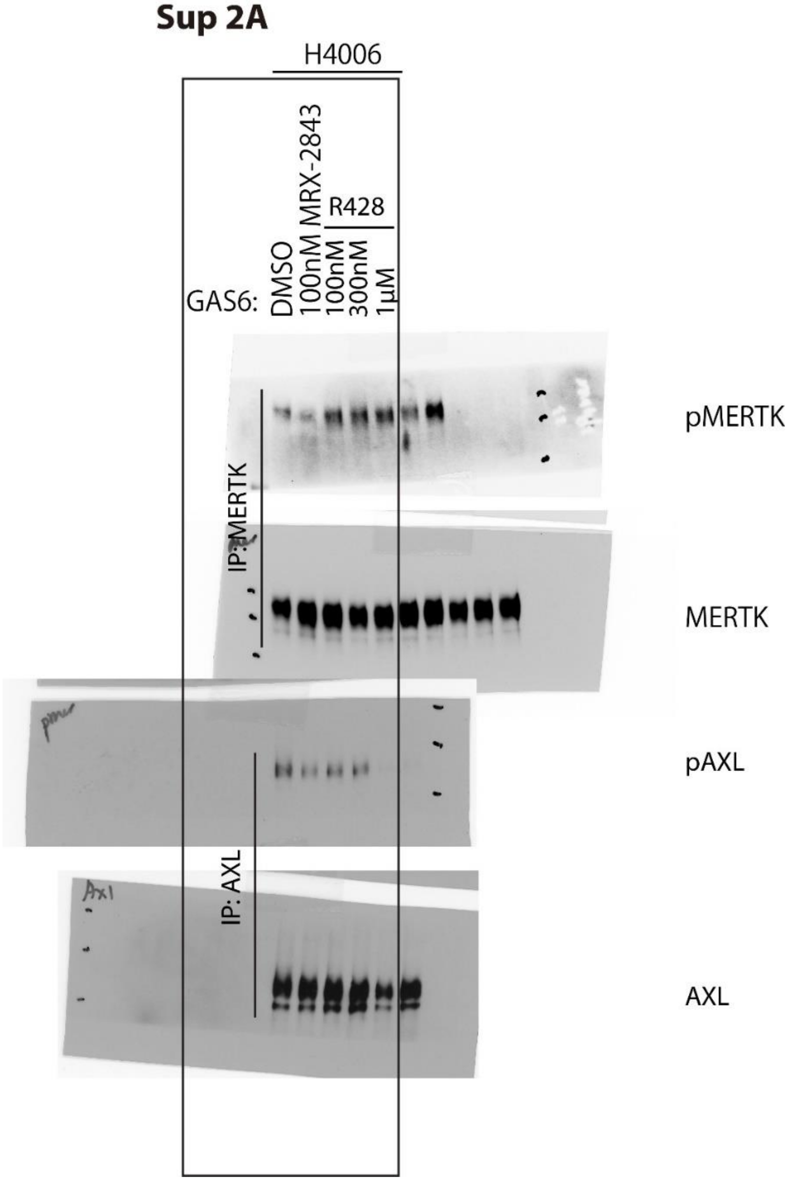

Supplement: Supplemental data [file jci-132-150517-s026.pdf]
